# Progress in antibacterial applications of nanozymes

**DOI:** 10.3389/fchem.2024.1478273

**Published:** 2024-09-23

**Authors:** Keyuan Zhao, Ye Zhao, Yuwei Wang, Bo Han, Meiling Lian

**Affiliations:** ^1^ Tianjin Engineering Research Center of Civil Aviation Energy Environment and Green Development, School of Transportation Science and Engineering, Civil Aviation University of China, Tianjin, China; ^2^ Tianjin Fire Science and Technology Research Institute of MEM, Tianjin, China

**Keywords:** nanozymes, enzyme-like activity, bacterial infections, antibacterial mechanism, broad-spectrum antibacterial properties

## Abstract

Bacterial infections are a growing problem, and antibiotic drugs can be widely used to fight bacterial infections. However, the overuse of antibiotics and the evolution of bacteria have led to the emergence of drug-resistant bacteria, severely reducing the effectiveness of treatment. Therefore, it is very important to develop new effective antibacterial strategies to fight multi-drug resistant bacteria. Nanozyme is a kind of enzyme-like catalytic nanomaterials with unique physical and chemical properties, high stability, structural diversity, adjustable catalytic activity, low cost, easy storage and so on. In addition, nanozymes also have excellent broad-spectrum antibacterial properties and good biocompatibility, showing broad application prospects in the field of antibacterial. In this paper, we reviewed the research progress of antibacterial application of nanozymes. At first, the antibacterial mechanism of nanozymes was summarized, and then the application of nanozymes in antibacterial was introduced. Finally, the challenges of the application of antibacterial nanozymes were discussed, and the development prospect of antibacterial nanozymes was clarified.

## 1 Introduction

There are thousands of species of bacteria that live in all possible environments around the world. Bacteria, as one of the main pathogenic microorganisms, can induce many kinds of diseases. These diseases are self-limiting, difficult to treat, and even highly fatal. At present, bacterial infection has become an increasingly serious problem, posing a major threat to global health, millions of people every year because of bacterial infections suffer and even die (Niu et al., 2021). Antibiotics are commonly used to fight against bacterial infections. The main mechanisms of antimicrobial action are inhibition of cell wall synthesis, interaction with cell membrane, interference with protein synthesis and inhibition of nucleic acid replication and transcription (Xie et al., 2023). However, the overuse of antibiotics and the evolution of bacteria led to the emergence of drug-resistant bacteria, and the therapeutic effect has been seriously reduced (Sun et al., 2022). Therefore, people are committed to developing new effective antibacterial strategies and designing new generation of antibacterial drugs to combat multi-drug-resistant bacteria and bacterial infections.

In recent years, nanomaterials play an important role in the field of antibacterial because of their unique physical and chemical properties (Gupta et al., 2019). Compared with antibiotics, nanomaterials can be designed by functional requirements, and their antibacterial effects can be regulated by controlling their size (Wang W. et al., 2020), morphology and structure (Wang T. et al., 2021), and by surface modification (Chen G. et al., 2021). And nano-antibacterial materials have good membrane permeability, can achieve high-efficiency antibacterial through multiple mechanisms, and are not easy to cause bacterial resistance (Gupta et al., 2019). Furthermore, the synthesis of nano-antibacterial materials is relatively straightforward, which contributes to their low production costs and minimal toxicity. However,it is important to acknowledge the challenges associated with these materials, such as their limited specificity, selectivity,and biocompatibility, which are areas that require ongoing research and development to optimize their therapeutic potential.

Nanozyme is a kind of nanomaterials with enzyme-like catalytic activity (Hou and Xianyu, 2023). It has unique physical and chemical properties of nanomaterials and high catalytic activity of natural enzymes. Although natural enzymes have high catalytic activity, high substrate selectivity and good biocompatibility, their high production cost, difficult storage and low catalytic stability make them less practical (Liu Y. et al., 2022). In contrast, nanozymes have the advantages of designability, diverse structure, easy multi-function, adjustable catalytic activity, high stability, low cost, easy storage and large production capacity. It has been widely used in many fields, such as biosensing (Huang et al., 2019; Zhang L. et al., 2021), immune analysis (Song G. et al., 2023), *in vivo* imaging (Zhao et al., 2023), disease diagnosis and treatment (Fan et al., 2023). In addition, the enzyme-like activity of nanozymes can catalyze the production of reactive oxygen species (ROS), too much ROS can damage bacterial cell membranes, destroy cell active substances, and kill bacteria (Tang et al., 2023). Therefore, nanozymes have shown a broad application prospect in the field of antibacterial. So far, a large number of nanomaterials such as metal-based (Liu H. et al., 2023), carbon-based (Wen et al., 2024), metal oxides (Yu B. et al., 2020), metal sulfides (Gao et al., 2021), metal-organic frameworks (MOFs) (Xie et al., 2020), MXene (Dai et al., 2023), etc., have been used to develop nanozymes. It has been found that nanozymes have high broad-spectrum antibacterial activity and can be used to treat bacterial infection *in vitro* and *in vivo*. In addition, there are a lot of researches that combine the catalytic activity of nanozymes with photothermal therapy (PTT) (Jin X. et al., 2024), photodynamic therapy (PDT) (Xi et al., 2021), chemical dynamic therapy (CDT) (Cao et al., 2022), sonodynamic therapy (SDT) (Yang et al., 2020), etc. These strategies can not only improve the catalytic activity of nanozymes, but also achieve better antibacterial effect.

**FIGURE 1:**
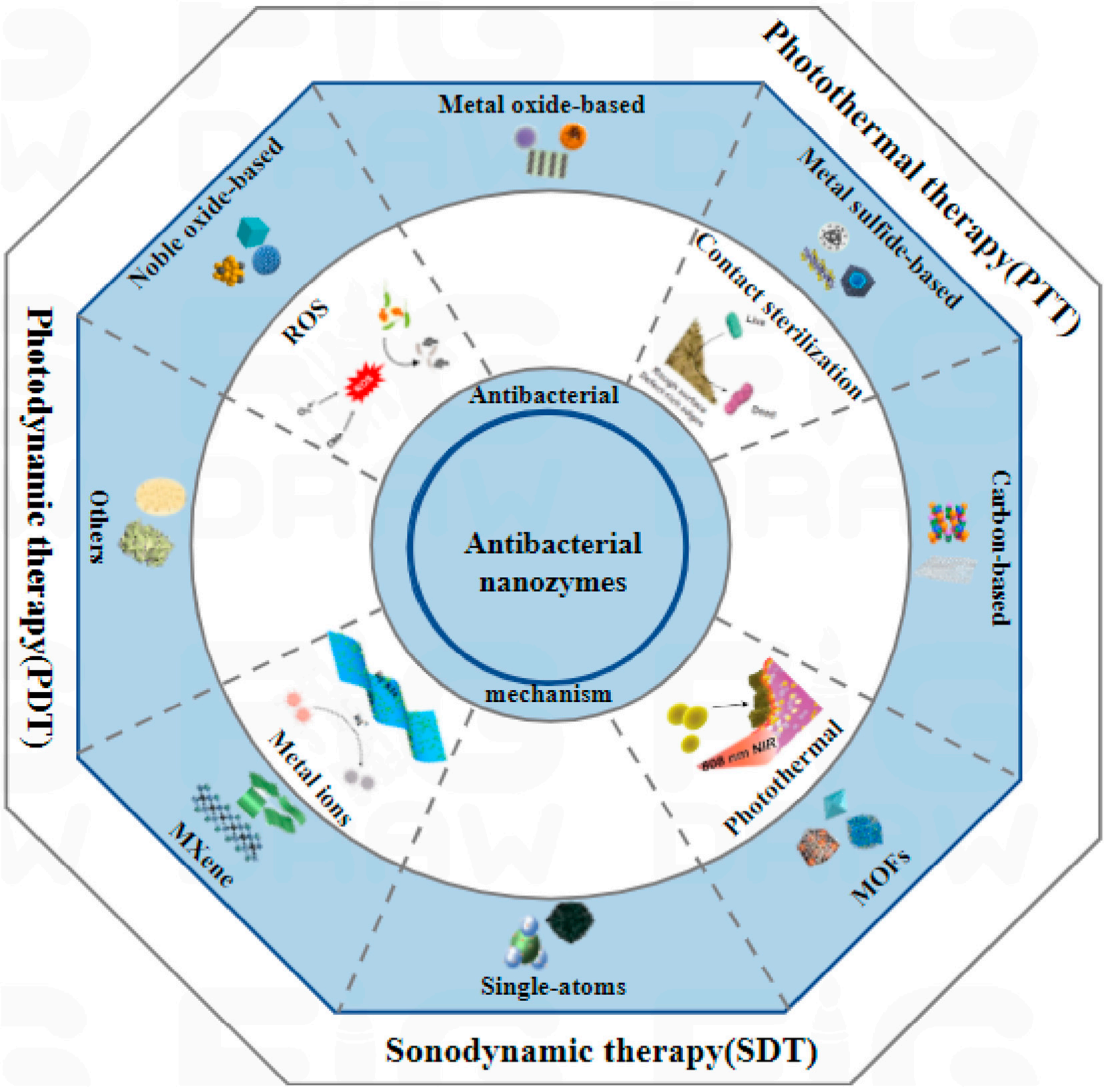
Schematic diagram of different kinds of nanozymes and their antibacterial mechanisms.

In this review, we mainly introduce the application of some kinds of nanozymes in antibacterial field in recent years (Figure 1). Firstly, the possible antibacterial mechanism of nanozymes was introduced. Secondly, the antibacterial activity and application of a series of nanozymes were introduced. And then, the research of nanozymes combined with other antimicrobial therapy to enhance the performance was also described. Finally, the future needs to pay attention to the problems and challenges of the outlook are discussed.

## 2 Antibacterial mechanism of the nanozymes

Nanoenzymes, as an emerging nanomaterial, are gaining attention from researchers for their application in the antibacterial field. The antimicrobial efficacy of nanoenzymes is mediated through multiple mechanisms, mainly including reactive oxygen species generation(ROS) (Zhang D. et al., 2023), contact sterilisation (Liu et al., 2021), metal ion antimicrobial (Yao et al., 2023), and photothermal antimicrobial (Xu et al., 2019). Given that different types of nanoenzymes can have different structural, physical and chemical properties as well as catalytic abilities, effective killing of different bacteria can be achieved by precisely modulating the size, surface modification and active centre of the nanoenzymes.

First, the production of ROS is the main antibacterial mechanism of nanozymes (Zhang Y. et al., 2023). ROS includes superoxide anion (O_2_^.−^), hydrogen peroxide (H_2_O_2_), hydroxyl radical ( OH), and singlet oxygen (^1^O_2_), etc., which are natural by-product of normal oxygen metabolism and play an important role in cell signaling and homeostasis. Nanozymes with oxidase or peroxidase activity can catalyze the oxidation of molecular oxygen and H_2_O_2_ to generate ROS. ROS can interact with bacterial cells, damage cell walls and membranes, degrade DNA, destroy active structures such as proteins, nucleic acids and lipids, and ultimately lead to bacterial death (Jiang et al., 2024).

Second, contact sterilization is also the antibacterial mechanism of nanozymes (Liu et al., 2021). The negatively charged bacteria attract positively charged nanozymes, and penetrate the bacterial membrane through the transfer of positive and negative charges, causing bacterial death. Besides, the sharp edges and edges of the nanozymes contact with the bacteria will puncture the cell membrane, causing damage to the cell membrane, the loss of nutrients within the bacteria, seriously destroy the bacteria and even cause bacterial death. The interaction of the nanozymes itself with the bacterial cell causes the bacteria to stick to the surface of the nanozymes, trapping the bacteria in the damage zone and interfering with the transport of electrons, ions and nutrients that affect the growth of the bacteria, cause the bacteria to die.

Next, metal ions also play an important role in the antibacterial activity of nanozymes (Yao et al., 2023). Some nanozymes also release metal ions, such as silver ions (Ag^+^), copper ions (Cu^2+^), zinc ions (Zn^2+^), and iron ions (Fe^2+^). These metal ions can pass through the cell membrane of bacterial to destroy the material in the cell, leading to the inactivation of key components of the bacterial function. It has good germicidal effect to many kinds of pathogenic bacteria.

Finally, photothermal antibacterial is also a hot spot in the field of antibacterial (Xu et al., 2019). Nanozymes that possess photothermal capabilities are particularly noteworthy; they have the innate ability to convert absorbed light into heat. When these nanozymes are exposed to near-infrared light, which is characterized by its deep tissue penetration and favorable biocompatibility, they can generate localized high temperatures. This heat can effectively disrupt the lipid bilayer of bacterial membranes, leading to the leakage of cellular contents and, ultimately, the death of the bacteria.

Nanozymes combine the efficient catalytic properties of enzymes with the specific targeting properties of nanomaterials. However, the exploration of specific targeting mechanisms is indeed a complex area, as different nanozymes may act against different structures or metabolic pathways in bacteria (Villalba-Rodríguez et al., 2023). The specific targeting mechanism of nanozymes relies on the following aspects:

(1) Size and shape: The size of nanozymes can be precisely controlled, which allows them to be localised directly in specific tissues or cells within an organism. For example, nanoparticles may carry specific ligands or coupled ligands that can be targeted by binding to receptors on cell membranes (Jana et al., 2024).
(2) Ligands or antibodies: Nanozymes may be labelled with functional ligands or antibodies that can bind to specific targets (e.g., antigens, receptors or specific chemical molecules) to achieve targeting (Gao et al., 2021).
(3) Physicochemical properties: The surface properties of nanozymes, such as electrical charge, magnetic or optical properties, can be utilised to direct them to specific locations. For example, through magnetic guidance, they can be delivered to a target area under the action of a magnetic field (Stasyuk et al., 2020).
(4) Drug carrier: Nanozymes can be used as carriers for drugs or growth factors to achieve targeted therapy through controlled release (Zhang et al., 2024).
(5) Biocompatible: Nanozymes designed to be biocompatible can increase targeting effectiveness by reducing immune reactivity and improving stability in specific microenvironments (Zhu et al., 2022).

Nanozymes have demonstrated the diversity and effectiveness of their targeting mechanisms in different application scenarios. They can achieve precise targeting of specific cells or tissues through surface functionalization modifications, such as antibodies, antigens, cell membrane ligands, etc., as well as the interaction of external stimuli, such as magnetic fields, light, etc., with nanozymes. In the field of biomarkers and imaging, nanozymes can be used in magnetic resonance imaging (MRI) or fluorescence microscopy imaging by labelling specific targeting molecules, such as antibodies or antigens, especially in cancer detection (Zhao et al., 2020). In drug delivery, nanozymes, as carriers, can carry drugs to the site of disease and selectively adsorb to the surface of tumour cells by targeting design, increasing the local concentration and therapeutic efficacy of drugs (Liu Y. et al., 2022). In addition, certain nanozymes can specifically catalyse the release of drugs, such as under light, heat or magnetic field activation, to achieve precise treatment of tumours (Yu Z. et al., 2020). In environmental remediation, the catalysis of nanozymes can be used to decompose harmful chemicals in the environment and improve environmental pollution (Diao et al., 2024). As active components of biosensors, nanozymes show high sensitivity and selectivity in detecting biomarkers such as blood glucose and urea (Wang G. et al., 2023). At the same time, nanozymes can also be designed as controlled release systems that release under specific conditions such as pH or temperature changes, improving the precision of the application (Mei et al., 2021). These versatility and effectiveness make nanozymes promising for biomedical and environmental applications.

## 3 Antibacterial nanozymes

After detailing the antimicrobial mechanism of nanoenzymes, the antimicrobial nanoenzymes are investigated in this section. According to relevant literature, various research teams are committed to exploring the applications of nanozymes with enzyme-like activities in a multitude of fields, such as catalysis (Li W. et al., 2023), biosensing (Song N. et al., 2023), disease diagnosis and treatment (Xi et al., 2019),etc.,.In addition, nanozymes with enzyme-mimicking catalytic activities have shown superior broad-spectrum antimicrobial properties, capable of efficiently and effectively killing bacteria. Theyalso possess excellent biocompatibility, allowing for the rapid and effective treatment of bacterial infections without the risk of inducing resistance.In the following, we will categorize anti-bacterial nanozymes based on the materials used in a straightforward manner.

### 3.1 Noble metal-based nanozymes

The current study shows that some noble metal nanozymes, such as gold, silver, platinum, palladium, rhodium (Au, Ag, Pt, Pd, Rh) etc., have good enzyme-like activity and excellent antibacterial activity. In addition, it is also found that the hybrid nanozymes containing noble metals also has excellent antibacterial properties and has been widely used. Table 1 summarizes the antimicrobial applications of some noble metal-based nanozymes with enzyme-like catalytic activity.

**TABLE 1:** Application of noble metal-based nanozymes in antibacterial.

Nanozymes	Enzymatic activity	Targets	Antibacterial mechanisms	Applications	References
Au NCs	OXD, POD	*E. coli*, *S. aureus*, MDR *E. coli*, MRSA, MDR A. baumannii, MDR *K. pneumoniae*, MDR *P. aeruginosa*, VRE, MRSA biofilms	Generate ROS	Clinical application of multidrug-resistant superbacterial infections	Zheng et al. (2018)
BGN-AuNCs	POD	*E. coli*, *S. aureus*	Generate OH, super charge	Treat infectious diseases and accelerate regeneration	Xu et al. (2022)
DAPT-AuNCs	—	*E. coli*, *S. aureus*, MDR *E. coli*, MRSA	Destroy membrane integrity, generate ROS	Prevent and treat skin infections caused by a variety of bacteria	Xie et al. (2021)
Ag NCs	POD	*P. aeruginosa*, MDR *P. aeruginosa*	Membrane damage, generate ROS	Treatment of multi-drug resistant *P. aeruginosa* infection	Chen et al. (2020)
AgNPs-AMP@PSiMPs	—	*E. coli*, *S. aureus*	Release of Ag^+^, release of AMP	Treat wound infection and wound healing	Jin et al. (2021)
rAgNAs	—	MRSA, MRSA biofilm	Release of Ag^+^	Treatment of drug-resistant bacterial biofilm-associated infectious diseases	Wu et al. (2019)
Pt-Fmoc-FF hydrogel	OXD, POD	*E. coli*, *S. aureus*	Generate·OH and O_2_^·−^	Change the pH limit of nanozymes, accelerate the clinical application of nanozymes antibacterial therapy	Chen P. et al. (2022)
APGH	POD	*S. aureus*	Generate OH	Regulation of local microenvironment to promote the biological application of nano-enzyme	Chen G. et al. (2021)
Ag@Pt nanozymes	POD	*E. coli*, *S. aureus*	Generate ROS	Treat bacterial infections	Du et al. (2023)
Au/Pt NCs@GO_X_	POD	F. nucleatum	Generate OH	Treat oral disease	Wang Y. et al. (2023)
BiPt@HMVs	OXD, POD	CRE, MRSA	Generate ROS, US	Clinical treatment of multi-drug resistant bacteria-induced infection	Yao et al. (2023)

**FIGURE 2:**
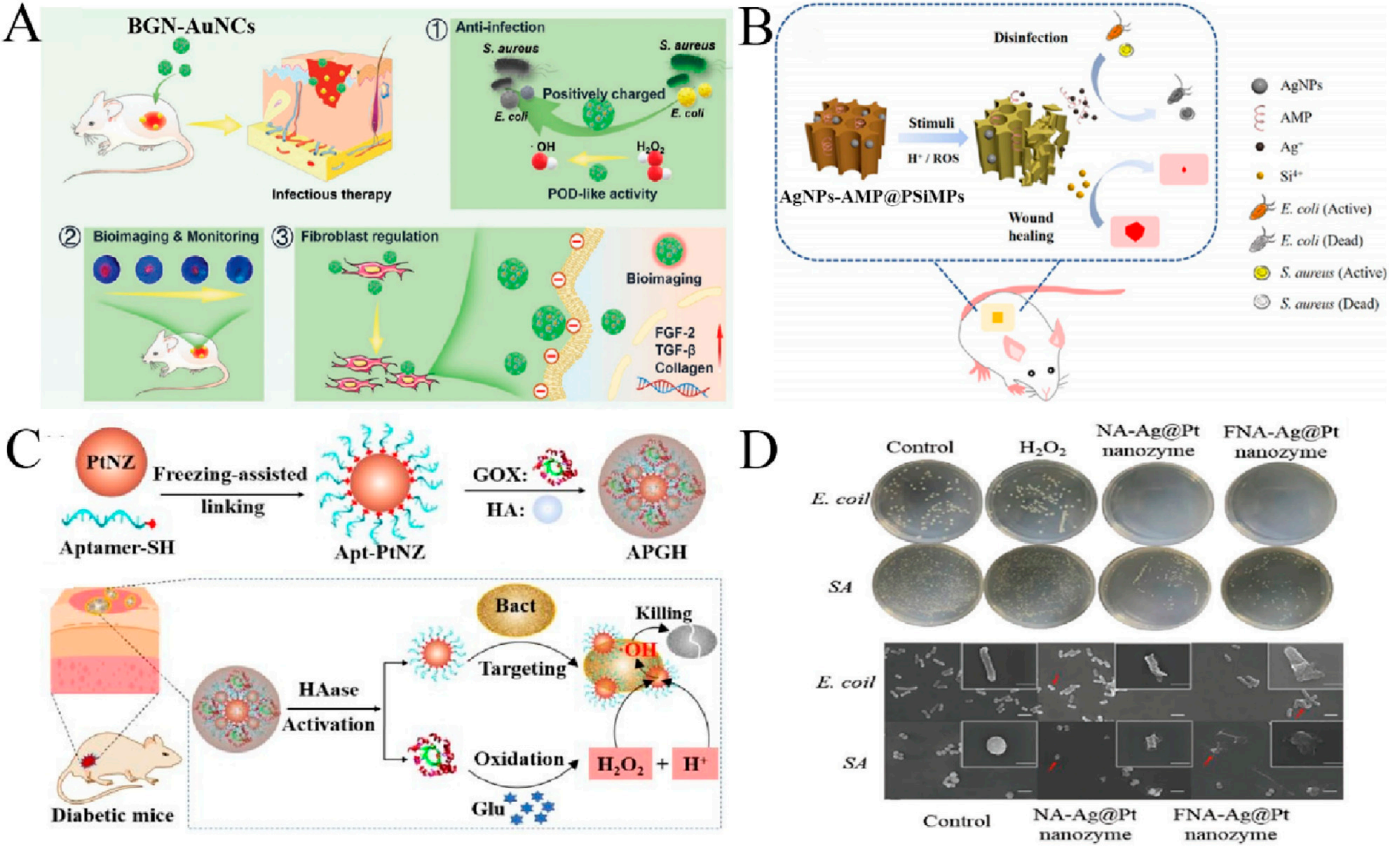
**(A)** Application of BGN-AuNCs in the treatment of infected wounds (Xu et al., 2022). **(B)** Antibacterial AgNPs-AMP@PSiMPs for wound disinfection and healing (Jin et al., 2021). **(C)** Antibacterial application based on APGH (Chen L. et al., 2021). **(D)** Antibacterial effect of Ag@Pt nanozymes (Du et al., 2023).

Gold nanoparticles (Au NPs) have stable chemical property, good biocompatibility and low environmental toxicity. However, its inherent stability and inertia, as well as low catalytic activity, limit its ability to achieve effective antimicrobial therapy. While controlling its size to nanocluster (NC) size (usually less than 2 nm), ultra-small Au nanoclusters (Au NCs) display surprisingly high and broad-spectrum antimicrobial activity based on their unique structure and physicochemical properties (Zheng et al., 2018; Zheng et al., 2021). Zheng et al. (2018) demonstrated for the first time that the broadspectrum antibacterial properties of Au NCs are attributable to its intrinsic oxidase (OXD) and peroxidase (POD) catalytic activity. In this study, four thiopyrimidine analogues were used as ligands to synthesize Au NCs. It was found that Au NCs could effectively destroy bacterial cell membranes by electrostatic adsorption, changes in cell membrane permeability make it easy for Au NCs to internalize into bacterial cells, inducing genomic DNA damage. More importantly, the intrinsic oxidase and peroxidase catalytic properties of Au NCs enable them to strongly induce intracellular ROS production upon entry into cells, thus accelerating bacterial death. Au NCs is also effective against resistance and has good biocompatibility.In addition, Au NCs also has very effective antimicrobial activity, which can significantly inhibit the formation of bacterial biofilms. Finally, through skin infection model and mouse pneumonia model, we found that Au NCs has great potential in the clinical application of multi-drug resistant superbacterial infection. After that, Wu et al. (2019) and Mei et al. (2021) have also synthesized ultra-small size Au NCs, and studied their killing behavior on Gram-negative and Gram-positive bacteria to clarify the antibacterial mechanism of Au NCs. Based on the supercharged properties of Au NCs, Xu et al. (2022) developed a novel strategy for the design of bioactive glass nanoparticles (BGN) using supercharged gold nanoclusters (AuNCs) with enhanced enzyme mimicry activity, can effectively fight bacterial infection and promote tissue regeneration (Figures 2A, B). Functional AuNCs made BGN possess excellent peroxidase activity and catalytic bacteriostasis. Due to the production of highly toxic OH, BGN-AuNCs can rapidly kill bacteria at a low dose (75 μg mL^−1^) within 6 h. The strong electrostatic interaction between the positive BGN-AuNCs and the negative bacteria was also proved to be the contact killing effect. The results of treatment of infectious wounds also confirmed the remarkable ability of BGN-AuNCs to treat infectious diseases and accelerate regeneration. In addition, through the rational design of AuNCs, the elimination of a variety of bacteria can also be achieved. Xie et al. (2021) developed 4,6-diaminino-2-pyrimidinethiol(DAPT)-modified AuNCs (DAPT-AuNCs) against Gram-negative and Gram-positive bacteria strains as well as their MDR counterparts. It also has good biocompatibility, and its broad-spectrum antibacterial activity has a good preventive and therapeutic effect on skin infections caused by various unknown bacteria. Pang et al. (2021) developed dual-ligand-functionalised Au NCs to obtain Au NCs with excellent antibacterial ability and high stability, which achieved high antibacterial activity against Gram-positive MDR bacteria. Zhang et al. (2024) used UV-nanoimprint lithography (UV-NIL) associated with the glancing angle deposition (GLAD) process of electron beam evaporation to prepare Au-coated PLGA nanocylinders loaded with Paclitaxel (PTX) (PTX-PLGA-Au NCs) to enhance anticancer efficacy by the cooperative treatment of photothermal-chemotherapy.PTX-PLGA-Au NCs with different length-to-diameter ratios can be prepared by controlling the concentration of Poly(Lactic-co-Glycolic Acid) (PLGA) and regulating the angle of GLAD. Research shows that PTX-PLGA-Au NCs exhibit a good photothermal effect. The high temperature generated by the Au layer on their surface under laser irradiation promoted the rapid release of PTX and realised the synergy of photothermal chemotherapy, thus effectively developing a multi-functional nano-platform for cancer treatment.

Silver-based nanozyme has strong antibacterial properties, and its antibacterial mechanism is mainly through the dissolution and release of Ag^+^. It can also induce the production of ROS in cells, destroy bacteria and cell membranes, and ultimately kill bacteria (Martorana et al., 2022). Because of the excellent performance of Ag NPs in antibacterial, people have been devoting themselves to exploring the antibacterial mechanism of Ag NPs, and there are many researches on its antibacterial application (Stabryla et al., 2021). Ag NPs has a size-dependent antibacterial efficiency, and the smaller the size of Ag NPs, the stronger the antibacterial ability (Dogru et al., 2017). Pucelik et al. (2022) synthesized functional Ag NPs using a low-cost, environmentally friendly sodium borohydride process. Ag NPs maintains excellent stability and exhibits unprecedented properties as antibacterial and anticancer agents. ROS production and impairment of the cellular redox state enable Ag NPs to easily cross the cell membrane and interact with DNA, leading to apoptosis and ultimately cell death. The results show that Ag NPs with multiple potential targeting activities can be greatly utilized to control various diseases, including cancer and multi-drug resistant bacteria. Some researchers have further studied silver nanoclusters (Ag NCs) with ultra-small size as antibacterial materials (Chai et al., 2023). For example, Chen et al. (2020) have successfully synthesized amphiphilic Ag NCs, which can more easily penetrate the outer membrane of bacteria, causing membrane damage and enhancing the possibility of interaction between Ag NCs and biomolecules in bacterial cells. In addition, Ag NCs have strong peroxidase activity and the ability to promote the generation of ROS, showing excellent antimicrobial properties against MDR P.aeruginosa. It has good biocompatibility and also has favorable therapeutic effects for pneumonia mice caused by P.aeruginosa. In any case, the release of Ag^+^ is the main reason for the antibacterial activity of silver-based nanomaterials. However, excessive release of Ag^+^ may have serious toxic effects on normal tissues. In order to reduce the cytotoxicity of Ag^+^, Ag NPs were combined with other antibacterial agents to enhance the antibacterial effect. Using porous silicon (PSi) as a carrier, loaded with Ag NPs and antimicrobial peptide (AMP), a dual synergistic antibacterial platform (AgNPs-AMP@PSiMPs) with on-demand release capability was developed by Jin et al. (2021). The combination of AgNPs and AMPs showed good synergistic antibacterial effect. As shown in Figure 2B, due to oxidative and desorption effects, AgNPs-AMP@PSiMPs has good on-demand release behavior under acid and ROS stimulation, and is able to control the release of Ag^+^ and AMP upon wound infection, with strong bacteriostatic effect and lower cytotoxicity, promote wound healing and cure drug-resistant bacterial infection. Falsal et al. (2024) synthesized Pt NPs from *Enterobacter cloacae* in patients with urinary tract infection.They next created amide linkages to load Resveratrol (RSV) onto Bovine Serum Albumin (BSA)-conjugated Pt NPs and release it into cancer cells’ acidic environment. They also tested Pt-BSA-RSV-NPs against different other biological properties, such as; anti-bacterial, anti-oxidant, and anti-inflammatory properties.This research work finds that the designed nanocomposites have good biocompatibility and therapeutic potential.

In addition to the common Au and Ag nanoenzymes, there has been an increasing number of studies based on Pt nanoenzymes, which have been found to have good catalytic activity and cellular bactericidal capacity The optimal activity of most nanozymes occur at an acidic pH, whereas in biological systems the pH exceeds 7.0 and even exceeds 8.0 in chronic trauma. To improve the antibacterial activityof H_2_O_2_ and avoid the toxicity of high level H_2_O_2_, Chen et al. (2022b) encapsulated Pt NPs into 9-fluorenylmethoxycarbonylmodified diphenylalanine (Fmoc-FF) hydrogel by self-assembly, and Fmoc-FF hydrogel provided an acidic microenvironment for Pt NPs. After encapsulated in Fmoc-FF hydrogel, Pt NPs exhibited a 6-fold enhan-ced OXD-like activity and a 26-fold POD-like activity with good synergistic antibacterial effect. This design p-rovides a strategy to break the pH limit of nanozymesand promotes the biological application of nanozymes. Chen L et al. (2021) constructed an activatable nanozyme (APGH) with CDT targeting capacity by cohousing aptamer-functionalized platinum nanozyme (Apt-PtNZ) and glucose oxidase (GO_X_) in a hyaluronic acid (HA) shell.As shown in Figure 2C, the nanozyme bind to bacteria through adaptor recognition, glucose oxidation lowers the pH of the bacterial surface, promotes peroxidase activity, and replenishes H_2_O_2_ to produce *in situ* ·OH on the bacterial surface. It can effectively promote the healing of infected wounds.

Compared to monometallic nanoenzymes, bimetallic nanoenzymes utilise the synergistic action of two different metal atoms and typically exhibit superior catalysis as well as better performance in antimicrobial properties. Through a one-step rapid self-assembly driven by nucleic acid and metal ion coordination, Du et al. (2023) designed and synthesized the Ag@Pt nanozymes with dual functions. An adjustable NA-Ag@Pt nanozyme was synthesized using a single-stranded nucleic acid sequence as a template, and then mixed with a functional nucleic acida (FNA) to produce an FNA-Ag@Pt nanozyme. Both Ag@Pt nanozymes all have powerful POD-like catalytic activity and antibacterial activity. The production of ROS by POD-like activity can effectively solve the problem of bacterial tolerance. Ag@Pt nanozymes have a broad spectrum of antimicrobial activity (Figure 2D) and is effective against bacterial infections. Wang G. et al. (2023) designed a bimetallic nanocluster enzyme (Au/Pt NCs) with high POD activity by doping Au NCs with Pt atoms, and then chemically coupled with glucose oxidase (GO_X_) to design a cascaded catalytic nanozyme Au/Pt NCs@GO_X_. GOx in the enzyme can convert low concentration glucose into gluconic acid and H_2_O_2_. The pH of the D-gluconic acid is regulated to acid, which is conducive to the POD-like activity of Au/Pt NCs@GO_X_ and the production of more ·OH, which plays an important role in the efficient antibacterial activity against F. nucleatum and the biofilm *in vitro*. *In vivo* and biosafety studies have shown that Au/Pt NCs@GO_X_ can be used to treat F. nucleatum-induced periodontitis.

### 3.2 Metal oxide-based nanozymes

In recent years, many studies have found that metal oxide nanozymes usually have broad-spectrum antibacterial activity and low biological toxicity, such as Fe_3_O_4_, CuO, CeO_2_, ZnO, Ag_2_O, TiO_2_, Co_3_O_4_, etc. In addition to single metal oxides, there are also some bimetal oxides. They can fight bacterial biofilms, eradicate different types of bacteria, even drug-resistant bacteria. Table 2 summarizes the antimicrobial applications of some metal oxide-based nanozymes with enzyme-like activity.

**TABLE 2:** Application of metal oxide-based nanozymes in antibacterial.

Nanozymes	Enzymatic activity	Targets	Antibacterial mechanisms	Applications	References
IONPs	POD	*E. coli*, *S. aureus*	Generate OH	Clinical anti-infective treatment	Yu B. et al. (2020)
PDA/Fe_3_O_4_	POD	*E. coli*, *S. aureus*	Generate OH, PTT	Effective treatment of bacterial infections	Xiao et al. (2022)
rough C-Fe_3_O_4_, RCF	POD	*E. coli*, *S. aureus*, MRSA	Generate OH, PTT, CDT	Effective treatment of drug-resistant bacterial infections	Liu et al. (2021)
Cu_x_O-PDA	POD	*E. coli*, *S. aureus*	Generate ROS	Clinical antibacterial	He et al. (2022)
CeO_2_/GOx nanoreactor	POD	*E. coli*, *S. aureus*	Generate OH	Biosensors, treatment and environmental repair	Qin et al. (2021)
Cu-CeO_2_ SSE	POD	*E. coli*, MRSA	Generate ·OH	Accelerates wound healing during antioxidant and tissue healing processes	Jiang et al. (2018)
Cu_1.5_Mn_1.5_O_4_	OXD, POD, GSH-Px	*E. coli*, MRSA	Generate OH	Treat bacterial infections	Wu et al. (2022)
NiCo_2_O_4_	POD	*E. coli*, *S. aureus*	Generate ROS, Mechanical damage	Design new engineering antibacterial material	Song G. et al. (2023)
Cu-Fe_3_O_4_	POD	*E. coli*, MRSA	Generate ROS	Resistant to pathogen infection and wound healing	Jin J. et al. (2024)

**FIGURE 3:**
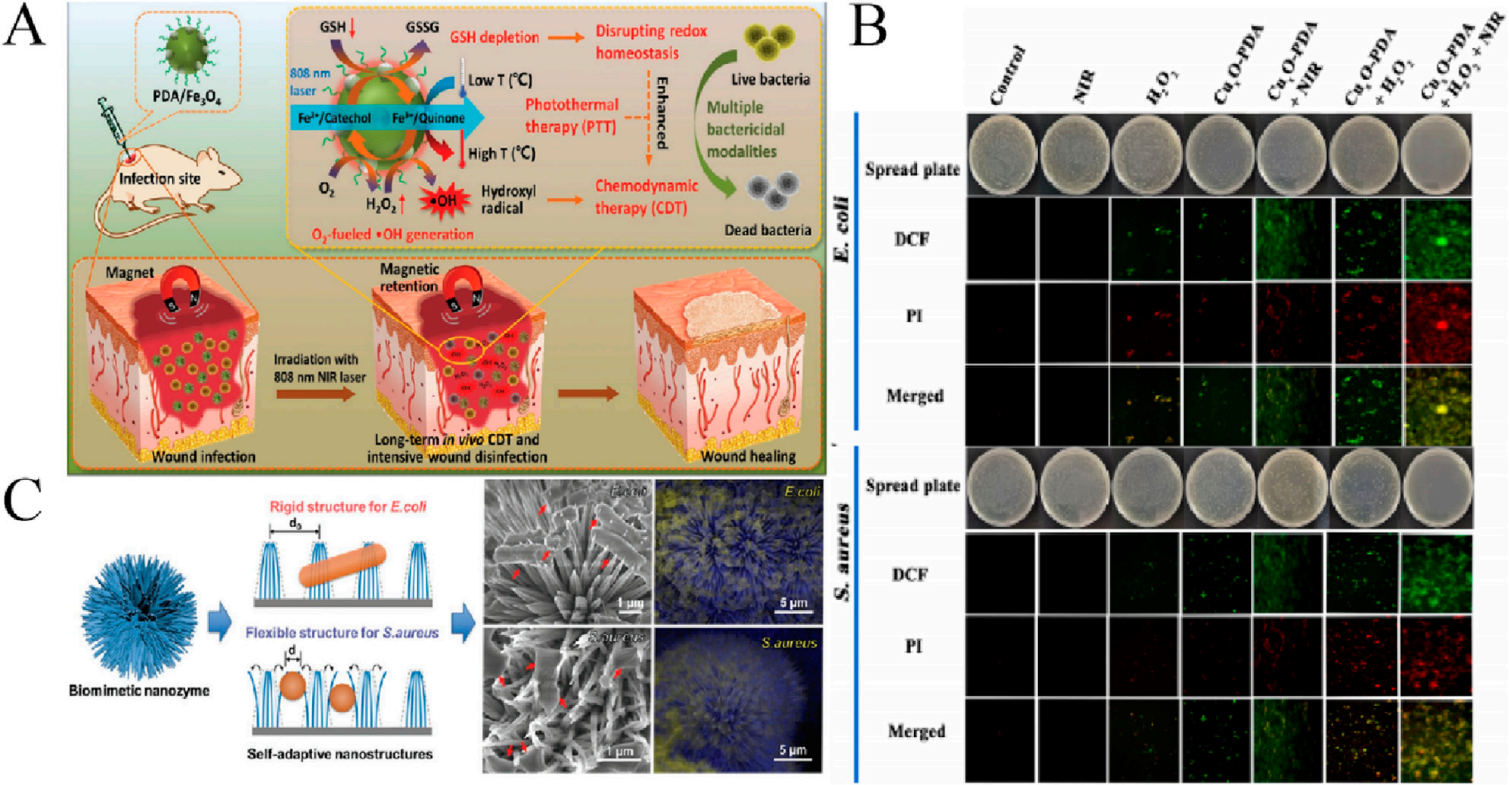
**(A)** The antibacterial nanozyme PDA/Fe_3_O_4_ was used in the treatment of infected wounds (Xiao et al., 2022). **(B)** Antibacterial effect of Cu_x_O-PDA (He et al., 2022). **(C)** Antibacterial effect of NiCo_2_O_4_ (Song G. et al., 2023).

Fe_3_O_4_ NPs are more stable than natural peroxidase. Fe_3_O_4_ NPs can catalyze H_2_O_2_ to produce highly toxic ·OH, and exhibit extremely high antibacterial activity against a variety of bacteria with the assistance of low concentrations of H_2_O_2_. Yu B. et al. (2020) showed that Iron oxide nanoparticles (IONPs) combined with Fenton-triggered strategies could stimulate the production of ROS by inducing polarization in M1 macrophages, significantly enhances the bactericidal effect of macrophages on intracellular *Staphylococcus aureus*. Xiao et al. (2022) constructed an intelligent nanozyme PDA/Fe_3_O_4_ which can consume GSH and supply H_2_O_2_ by mineralizing ultra-small Fe_3_O_4_ *in situ* on PEG-modified PDA. As showen in Figure 3A, the photothermal treatment of PDA/Fe_3_O_4_ nanozyme can not only destroy bacteria directly, but also improve the POD-like activity of Fe_3_O_4_ for CDT.In addition, the PDA/Fe_3_O_4_ nanozyme is able to deplete endogenous GSH to disrupt bacterial redox homeostasis through a photothermally enhanced cascade of catalytic reactions, while providing abundant H_2_O_2_ to promote ·OH production, finally, the antibacterial activity of CDT was enhanced. It showed good broad-spectrum antibacterial activity.

CuO NPs also has attracted attention due to its antimicrobial and biocide properties. CuO NPs has good biological properties, including effective antimicrobial activity against various pathogens and drug-resistant bacteria. Several reports showed that CuO NPs had significant antibacterial activity against different pathogenic microorganisms. Angeline Mary et al. (2019) synthesized CuO NPs by using SCJ as a stabilizer. The results showed that the positively charged bare CuO NPs could effectively adhere to the negatively charged bacterial cell wall through electrostatic interaction at neutral pH, therefore, it has obvious bactericidal activity against *E. coli*, *S. aureus*, *P. aeruginosa*, *Bacillus subtilis* and other bacteria. In contrast, the new functional CuO NPs have better catalytic and antibacterial activity than the bare CuO NPs. He et al. (2022) designed a novel copper oxide (Cu_x_O-PDA) nanozyme modified by polydopamine (PDA). The positive charge of Cu_x_O-PDA under acidic conditions can lead to targeted enrichment on the surface of bacteria, and the catalytic activity of nanozymes can be significantly enhanced by NIR radiation to produce a large amount of ROS, to further enhance its antibacterial activity (Figure 3B). Cu_x_O-PDA can damage the biological macromolecules (lipid peroxidation, DNA degradation) of bacteria, and has good antibacterial effect and good prospect of antibacterial application.

CeO_2_ nanomaterials are biocompatibility. As a kind of nanomaterials with multi-enzyme catalytic activity, it has attracted wide attention in the field of biological antibacterial. At the same time, due to its unique electronic configuration, namely reversible Ce^3+^/Ce^4+^ redox pair, CeO_2_ has strong antioxidant and pro-oxidative activity, which can scavenge reactive oxygen species. Zhao et al. (2020) designed a novel nanozyme, CeO_2_@MMT, which combined the multi-enzyme mimicking CeO_2_ NPs with montmorillonite (MMT), and CeO_2_ NPs gave it anti-inflammatory activity. MMT significantly reduced the systemic absorption of CeO_2_ NPs and reduced the toxicity of CeO_2_ NPs. CeO_2_@MMT specifically targets the inflamed colon through electrostatic interactions, scavenging ROS and reducing inflammation. Qin et al. (2021) constructed a nano-reactor of CeO_2_/GO_x_ by encapsulating glucose oxidase in mesoporous ceria hollow sphere nanozyme, showing excellent POD catalytic activity. The nano-reactor can produce gluconic acid to reduce pH value, thus effectively improve the catalytic activity of POD of CeO_2_ nanozyme, and catalyze the conversion of H_2_O_2_ molecule to highly toxic ·OH. It can effectively kill bacteria and prevent the formation of biofilm. Furthermore, *in vivo* experiments showed that CeO_2_/GOx was effective in eliminating 99.9% of bacteria in mouse wound tissues and preventing persistent inflammation without damaging normal tissues. In addition, the catalytic activity and antibacterial activity of CeO_2_ nanozymes can be improved by surface modification. Jiang et al. (2024) prepared the nanozyme Cu-CeO_2_ by modifying Cu units on CeO_2_ with various enzyme catalytic activities. The modification of Cu units effectively regulated the catalytic activity of CeO_2_ nanozymes. Compared with the original CeO_2_, Cu-CeO_2_ showed enhanced POD-like activity and decreased HORAC activity. *In vitro* and *in vivo* experiments showed that Cu-CeO_2_+H_2_O_2_ system successfully reversed the protective effect of CeO_2_ on bacteria and showed excellent antibacterial activity, which played an important role in the early anti-infection process.

In addition to single metal oxides, some bimetal oxide nanozymes have more efficient antibacterial activity. Wu et al. (2022) successfully synthesized bimetallic oxide Cu_1.5_Mn_1.5_O_4_ cage-like frame nanospheres (CFNSs) by two-step method of gas-assisted soft template solvothermal and calcination. Interestingly, Cu_1.5_Mn_1.5_O_4_ CFNSs showed enhanced triple enzyme activity: OXD, POD, and GSH-P_x_. Cu_1.5_Mn_1.5_O_4_ CFNSs with multi-enzyme activity showed significant combined antibacterial activity. In addition, *in vivo* experiments showed that Cu_1.5_Mn_1.5_O_4_ CFNSs could be conveniently used for wound disinfection. And it has good biological safety. After that, Jin J. et al. (2024) prepared Cu-Fe_3_O_4_ nanoclusters with enhanced multi-enzyme activity by a simple hot-solvent method for wound sterilization and wound healing. Cu-Fe_3_O_4_ nanoclusters bound H_2_O_2_ via POD and GSH-P_x_ activities to MRSA and *E. Coli* showed good antibacterial activity. Importantly, normal tissues are also protected from damage by ·OH and exogenous H_2_O_2_ through the synergistic action of SOD and CAT activity. The results of animal model experiment also proved that Cu-Fe_3_O_4_ nanocluster system can effectively eliminate MRSA infection and promote wound healing. Inspired by the nature and based on the idea of bionics, Song N. et al. (2023) developed a NiCo_2_O_4_ nanozyme with self-assembled three-layer nano-structure, which has excellent antibacterial activity. The nanozyme can capture various types of bacteria for mechanical destruction through the physical-mechanical interaction between nano-structure and bacteria (Figure 3C). In addition, the POD-like activity of the metal active site on the NiCo_2_O_4_ nanozyme can catalyze the oxidation to produce ROS, destroy the bacterial membrane and induce apoptosis. The nanozyme has a high antibacterial activity against both *E. coli* and *S. aureus* due to its mechano-catalytic coupling property. Guo et al. (2024) designed a natural non-toxic hydrogel loaded with bimetallic peroxide (CeCuO_x_) with H_2_O self-supply and synergistic effect mediated cascade of Fenton catalytic chemistry for efficient water disinfection. The CeCuO_x_ could *in situ* decompose to H_2_O_2_ and bimetallic oxide by responding to the weakly acidic water body, followed by direct ^.^OH generation via Ce/Cu synergistic-enhanced Fenton-like catalytic reaction, which significantly improved the antibacterial activity without any assistance of O_2_ and external energy. This study provides a simple efficient, low-cost and affordable path in the field of water disinfection.

### 3.3 Metal sulfide-based nanozymes

Nanomaterials derived from metal sulfides, including but not limited to molybdenum disulfide (MoS_2_), iron disulfide (FeS_2_), copper sulfide (CuS), and silver sulfide (Ag_2_S), have been the subject of extensive research and development efforts. This is primarily due to their remarkable properties, which have found particular utility in the realm of antimicrobial applications. Table 3 summarizes the antimicrobial applications of some metal sulfide-based nanozymes with enzyme-like activity.

**TABLE 3:** Application of metal-sulfide -based nanozymes in antibacterial.

Nanozymes	Enzymatic activity	Targets	Antibacterial mechanisms	Applications	References
MoS_2_ NSs	—	*E. coli*, *S. aureus*, MDR *E. coli*, MRSA	Generate ROS	Use solar energy to achieve efficient disinfection	Zhao et al. (2021)
R-CMs	POD	*E. coli*, *S. aureus*	Generate OH	Treat bacterial infections	Cao F. et al. (2019)
MoS_2_/CoS_2_ NFs	POD	*E. coli*, *S. aureus*, MDR *E. coli*, MRSA	Generate OH	Treatment of drug-resistant bacterial infections and wound healing	Wang Y. et al. (2023)
CS-MoS_2_	—	*E. coli*, *S. aureus*	Generate ROS, membrane damage	A potential bactericidal alternative with high antibacterial activity is provided	Cao W. et al. (2019)
CNSs@FeS_2_	—	*S. aureus*, *E. coli*, *S. typhimurium*, *P. aeruginosa*, S. mutants, M. albicans	Release of Fe^2+^	Wound disinfection	Xi et al. (2021)
FeS@PDA	POD	MRSA, DR *E. coli*, *P. aeruginosa*	Generate OH, PTT, CDT	Efficient and multifunctional therapeutic diagnosis of bacterial infections	Li A. et al. (2022)
CuS@GDY	POD	*E. coli*, *S. aureus*, MRSA	Generate ROS, PTT	Quick sterilization and wound disinfection	Bai et al. (2022)
BWOA NPs	—	*E. coli*, *S. aureus*	Generate ROS	Promote wound healing	Wei et al. (2022)

**FIGURE 4:**
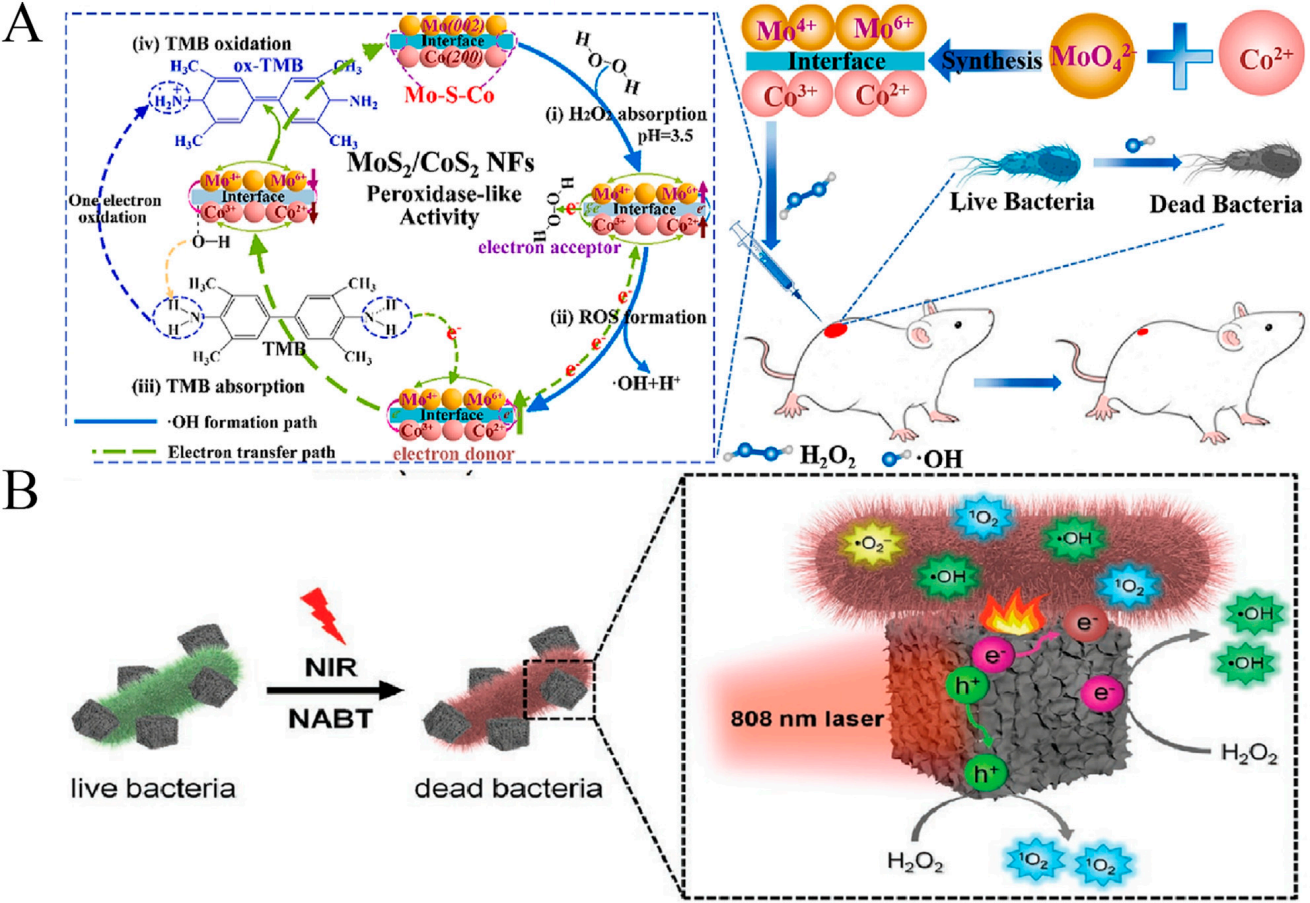
**(A)** Enzyme catalytic activity and antibacterial activity of MoS_2_/CoS_2_ NFs (Wang Y. et al., 2023). **(B)** Antibacterial mechanisms of CuS@GDY (Bai et al., 2022).

MoS_2_ is a typical two-dimensional transition metal dichalcogenides (2D TMDCs). It has many unique properties, such as high specific surface area, adjustable layer spacing, good photothermal effect, good biocompatibility, and easy surface functionalization. Based on this, MoS_2_ has a bright future in antibacterial applications with different antibacterial mechanisms. Zhao et al. (2020) found that MoS_2_ nanosheets (MoS_2_ NSs) had a strong effect on MDR bacteria under sunlight, and could inhibit the growth of MDR *E. coli* and MRSA reached the killing efficiency of over 99.9999%. Further mechanism research shows that the production of reactive oxygen species and the reduction of nanosheets’ size can improve the efficiency of solar energy disinfection. It is also proved that the size of MoS_2_ plays a very important role in the antibacterial properties of MoS_2_. More edges or defects on the surface of nanomaterials can provide more active sites for nanozymes, which can effectively improve the catalytic activity. Cao F. et al. (2019) developed a one-step method to construct a series of defective adhesion nanozymes with intrinsic bacterial capture and potent antibacterial properties.For example, MoS_2_ has excellent antibacterial effect because of its rough surface, abundant edge defects, high intrinsic POD-like activity and special strong bacterial adhesion properties. In addition, Wang G. et al. (2023) designed the MoS_2_/CoS_2_ heterostructure nanozymes using a simple molten salt method. The results show that the heterogeneous interface accelerates the electron transfer and enhances the activity of the enzyme. As shown in Figure 4A, MoS_2_/CoS_2_ has excellent broad-spectrum antibacterial activity based on its peroxidase activity, with a killing rate of up to 99% against resistant bacteria. In addition, MoS_2_/CoS_2_ also has good biocompatibility and promotes the rapid healing of wounds.There are many other metal sulphide nanozymes with enzyme-like activity, also have excellent antibacterial activity. The carbon nanospheres modified by ultrasmall FeS_2_ nanoparticles (CNSs@FeS_2_) were synthesized with hydrothermal method (Xi et al., 2021). CNSs@FeS_2_ releases both Fe^2+^ and S ions through dissolution and dissimilation. The release of Fe^2+^ leads to depletion of lipid peroxidation and GSH, which in turn causes degradation and death of bacterial DNA. Importantly, the released sulfur ions provide protection against Fe^2+^, ensuring the stable presence of Fe^2+^ for continued sterilization. In addition, the carbon shell of CNSs@FeS_2_ not only prevents FeS_2_ aggregation but also accelerates the release of Fe^2+^ through photothermal effects, enabling hyperthermia/Fe^2+^ synergistic treatment. This synergistic antibacterial system conveniently promotes internal wound disinfection. Bai et al. (2022) have developed a novel nanozyme based on Cu hollow sulphide nanocube coated with graphite diacetylene nanowire as a plasma enhanced nanozyme (CuS@GDY). The nanozyme had significant peroxidase catalytic activity and rapid, high-efficient and broad-spectrum antibacterial activity against a variety of pathogens. In addition, CuS@GDY showed enhanced catalytic activity when exposed to near-infrared light. It can also release a lot of heat. Therefore, the synergistic effect of photocatalysis and photothermal effect of nanozyme can effectively kill bacteria (Figure 4B). Li et al. (2024) synthesized a new cellulose-citrate-chitosan @ metal-sulfide nanocomposite (CL-CA-CS@CuS) through a one-pot reaction under mild conditions to develop an efficient adsorbent to remove water pollution.The prepared composite used intermittent technology to remove methyl orange (MO) dye from aqueous solution, and the composite also showed excellent efficiency against *S. aureus*. Wei et al. (2022) prepared Bi_2_Wo_6_-Ag_2_S direct Z-scheme heterostructure nanoparticles (BWOA NPs) by hydrothermal method. And it can accelerate wound healing. Under Sunlight Irradiation, BWOA NPs can produce abundant ROS due to its unique Z-type heterostructure, which has high antibacterial activity.

### 3.4 Carbon-based nanozymes

Carbon nanomaterials (CNMs) are renowned for their exceptional physical and chemical properties, which have made them a focal point of research across a multitude of disciplines. Among these are carbon nanotubes (CNTs), carbon dots (CDs), graphene oxide (GO), and carbon quantum dots (CQDs), each with its own unique characteristics and applications.The versatility of CNMs extends to the field of antibacterial applications, where they have demonstrated significant potential. Table 4, which is referenced here, elucidates the various ways in which carbon-based nanozymes contribute to the realm of antimicrobial technology.

**TABLE 4:** Application of Carbon -based nanozymes in antibacterial.

Nanozymes	Enzymatic activity	Targets	Antibacterial mechanisms	Applications	References
o-CNTs	POD	*E. coli*, *S. aureus*	Generate OH	Building new high-efficiency nanozymes to broaden the biological utility of o-CNT nanomaterials	Wang et al. (2018)
N-CNTs@Co	OXD	*E. coli*, *S. aureus*	Generate ROS	Effective treatment of bacterial infection wounds	He et al. (2021)
Fe-CDs	POD	*E. coli*, *S. aureus*	Generate ROS, PTT	Disinfection of wounds promotes healing	Liu Z. et al. (2022)
CS@Fe/CDs	POD	*S. aureus*, *P. aeruginosa*, biofilm	Generate OH, electrostatic interaction	Ensure food safety and environmental health	Pan et al. (2022)
Pt Co@Graphene	OXD	*E. coli*, *H. pylori*	Generate ROS	Clinical treatment of *Helicobacter pylori* infections	Zhang R. et al. (2021)
CuS/GO NC	OXD, POD	*E. coli*, *S. aureus*, MRSA	Generate OH	Resistance to multi-drug resistant bacterial infections	Wang W. et al. (2020)
GO-NTA-Ce	DNase	*S. aureus*, MRSA	Cut the biofilm	Treatment of drug-resistant bacterial biofilm infection	Hu et al. (2022)
FeLab	POD	C. albicans	Generate ROS	It provides a combined therapy of nanozyme and probiotics for the treatment of candidal vaginitis	Gen Wei et al. (2023)
PtRu/C_3_N_5_	OXD	*E. coli*, *S. aureus*, MRSA, *P. aeruginosa*	Generate ROS	Develop integrated antimicrobial and anti-inflammatory therapies	Shi et al. (2022)
CNQDs	POD	*E. coli*, *S. aureus*, *B. subtilis*, R. solani	Generate OH	Broad-spectrum antimicrobial agents in the fields of biomedicine and environmental protection	Li et al. (2018)
PdFe/GDY	POD	*E. coli*, *S. aureus*	Generate OH	Environmental and biomedical antimicrobial applications	Wang Y. et al. (2021)

**FIGURE 5:**
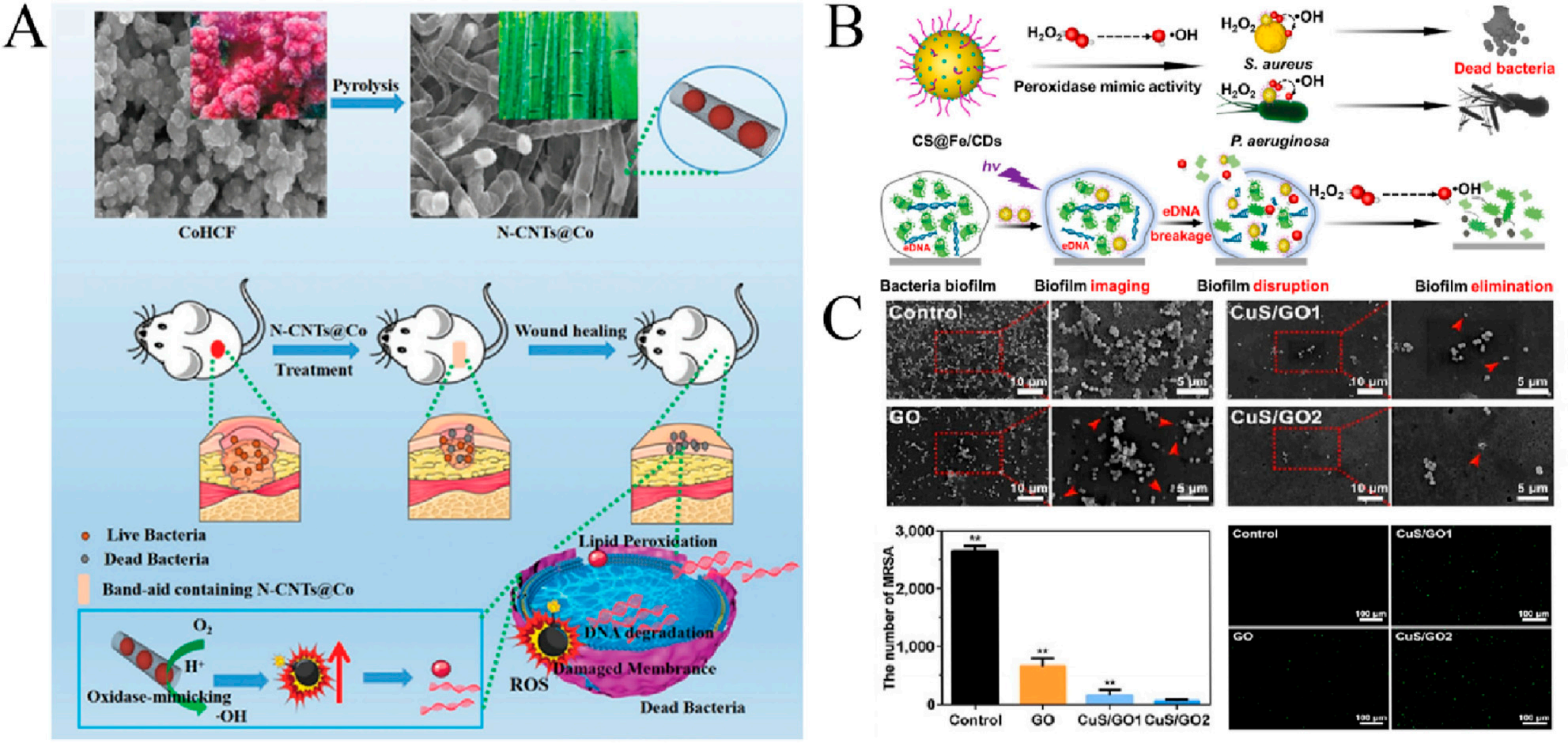
**(A)** Antibacterial application of N-CNTs@Co (He et al., 2021). **(B)** The peroxidase catalytic activity of CS@FeCDs nanozyme on bacteria and its elimination on bacterial biofilms (Pan et al., 2022). **(C)** The antimicrobial activity of CuS/GO NC (Wang W. et al., 2020).

Carbon nanotubes (CNTs) have high specific surface area, excellent chemical and thermal stability, and abundant electronic, optical and enzymatic properties. For the first time, Wang et al. (2018) have developed several o-CNTs rich in oxidation groups through one-pot oxidation reflux, and o-CNTs have high peroxidase activity over a wide pH range. The experimental results and theoretical calculations showed that the carbonyl group on o-CNTs surface is the active center, while the carboxyl group and the hydroxyl group act as the competitive center and inhibit the catalytic reaction, respectively. Furthermore, the carboxyl group showed stronger inhibition than the hydroxyl group due to its intrinsic hydrogen bond interaction and higher negative charge. O-carbon nanotubes (o-CNTs-BrPE) modified with 2-bromo-1-acetophenone were further prepared by deactivating the carboxyl groups present on o-CNTs surface, which had the highest peroxidase activity and biocatalysis efficiency. Based on this, o-CNTs can catalyze H_2_O_2_ to produce highly toxic hydroxyl group, which can inhibit bacterial infection and promote wound healing effectively. In addition, He et al. (2021) prepared bamboo like nitrogen-doped carbon nanotubes (N-CNTs@Co) by high temperature pyrolysis of cobalt cyanide. The artificial nanozyme has high-efficiency oxidase mimicking activity, and can catalyze oxygen to produce a large amount of ROS under acidic conditions. It has excellent antibacterial activity against both Gram-positive bacteria *S. aureus* and Gram-negative *E. coli*, and can treat bacterial infected wounds quickly and effectively (Figure 5A).

Carbon dots (CDs) is an important class of fluorescent carbon-based nanomaterials with small size, good chemicalstability and biocompatibility, and potential in the antibacterial field. Li et al. (2018) prepared low toxic and degradable CDs by one-step electrochemical method. It was found that even at low concentration, CDs showed strong broad-spectrum antibacterial activity and antifungal activity. And it can be completely degraded under visible light. The CDs also have enzyme-like activity to catalyze antibacterial activity. Liu et al. (2023a) prepared iron-doped carbon point nanozyme with excellent photo-thermal conversion and photo-enhanced enzyme-like properties by one-pot pyrolysis. Fe doping entrusts CDs with enhanced POD-like activity, resulting in the production of heat and ROS, which act in concert to effectively kill Gram-positive bacteria and Gram-negative bacteria, and promote wound healing, prevent infection. Importantly, the ultrastructure of Fe-CDs is biocompatibility and beneficial to clinical transformation (Liu Z. et al., 2022). Without the use of light, Pan et al. (2022) have designed a new type of active chitosan-grafted Fe-doped carbon dots(CS@Fe/CDs) artificial nanozyme. Its peroxidase catalytic activity enables CS@Fe/CDs to catalyze H_2_O_2_ to produce ·OH, while CS is able to bind negatively charged cell membranes via electrostatic action, the synergistic effect effectively destroyed the Gram-positive bacteria *S. aureus* biofilm and completely eliminated the biofilm of Gram-negative bacteria *P. aeruginosa* (Figure 5B). This strategy may provide a powerful method for the management of bacterial biofilm contamination in food safety and environmental protection. In addition, CDs and other metal or metal oxide nanozymes were hybridized to synthesize nanomaterials with excellent enzyme-like activity and antibacterial activity.

Graphene is a new material with a single-layer two-dimensional honeycomb lattice structure, which is composed of SP^2^ hybrid-bonded carbon atoms. It has excellent physical and chemical properties, stability and biocompatibility. It has been reported that graphene-based materials such as graphene, graphene oxide (GO), reduced graphene oxide (rGO) and graphene quantum dots (GQDs) have excellent enzyme-like activity and antibacterial activity, which have been widely used in antibacterial field. Zhang et al. (2021b) developed a super-stable pH-responsive graphite nanozyme, Pt Co@Graphene (Pt Co@G), which can be activated *in vivo*. In the acidic gastric environment, the OXD-like activity of Pt Co@G is activated and has good stability, catalyzes the production of ROS, and has excellent selective bactericidal activity. Pt Co@G@CPB nanozyme was prepared by modifying the bacterial binding molecule C18-PEGN-phenylboric acid on Pt Co@G, which can specifically target the *Helicobacter pylori* by increasing the local ROS concentration on the bacterial surface, significantly enhanced antibacterial activity. This stable graphite nanozyme may solve a key problem in the clinical treatment of *H. pylori* infections. A novel copper sulfide/graphene oxide nanocomposite (CuS/GO) was synthesized by a simple hydrothermal method (Wang Y. et al., 2020). CuS/GO exhibits excellent oxidase and peroxidase activity, effectively catalyzes H_2_O_2_ to produce toxic OH, and has a unique needle-like morphology capable of puncturing bacterial cell membranes. Therefore, both physical and chemical effects make CuS/GO NC have excellent antibacterial ability, which can effectively kill MRSA and other multi-drug resistant bacteria (Figure 5C), and promote wound healing of MRSA infection. Hu et al. (2022) reported that a multifunctional graphene-based nanozyme, GO-NTA-Ce, could eradicate drug-resistant bacterial infections. GO-NTA-Ce has Deoxyribonuclease (DNase)-like activity and excellent photo-thermal effect, as well as the inherent antibacterial activity of graphene. Therefore, the nanozyme can cleave the stubborn biofilm, kill the bacteria and effectively treat the infection of drug-resistant bacteria biofilm through the triple synergistic action. Gen Wei et al. (2023) developed a responsive hyaluronic acid (HA) hydrogel rGO@FeS_2_/*Lactobacillus*@HA (FeLab) by combining peroxidase rGO@FeS_2_ nanozyme with *Lactobacillus* that can produce lactic acid and H_2_O_2_, it has strong POD activity and antibacterial activity. FeLab treats candidal vaginitis and reduces its recurrence by regulating both the vaginal microenvironment and catalytic *Candida* albicans.

In addition, many other carbon-based nanomaterials with enzyme-mimicking properties are also widely used in the antibacterial field. Shi et al. (2022) designed the multi-vacancy graphitic carbon nitride C_3_N_5_ nanosheet (PtRu/C_3_N_5_) modified by Pt-Ru nanoalloy. It has piezoelectric-enhanced oxidase-like activity and photocatalytic hydrogen production. It has a high killing efficiency to the broad-spectrum bacteria in a short time, and has an obvious inhibitory effect on the inflammatory reaction after the visible light irradiation, which may accelerate the healing of the bacterial infected wound. Carbon nitride quantum dot nanozyme (CNQDs) with high nitrogen vacancy was prepared by a simple ultrasonic crushing method (Li et al., 2018). Experiments and Density functional theory (DFT) simulations show that the presence of NVS can alter the local electron distribution and prolong the π electron delocalization, thus enhancing the peroxidase activity. At the same time, CNQDs biocompatibility into the microbial interior by diffusion, improving the bacteria’s ability to bind, and enhancing the ·OH against the microbial accurate and rapid attack. CNQDs is highly effective against Gram-negative (*E. coli*), Gram-positive bacteria (*S. aureus*, *Bacillus subtilis*) and fungi (R. Solani). Wang T. et al. (2021) successfully prepared graphdiyne-supported PdFe nanosheets (PdFe/GDY) with intrinsic peroxidase activity by a simple hydrothermal method. PdFe/GDY is highly efficient at catalyzing H_2_O_2_ decomposition to produce ·OH, and it consumes Glutathione, which is surprisingly effective against bacteria. PdFe/GDY is an excellent biocompatibility for internal wound disinfection and healing.

### 3.5 MOFs-based nanozymes

The metal-organic framework (MOF), which consists of metal ions and organic ligands, is a kind of porous compound with crystal structure developed rapidly in recent years. Due to its diverse and adjustable structure, high specific surface area, controllable porosity and excellent chemical stability, MOFs have attracted much attention from researchers, there are many applications in the field of antibacterial research. Table 5 lists some of the applications of MOF-based nanozymes with enzyme-like activity in antibacterial field.

**TABLE 5:** Application of MOFs-based nanozymes in antibacterial.

Nanozymes	Enzymatic activity	Targets	Antibacterial mechanisms	Applications	References
V-POD-M	POD	*E. coli*, *S. aureus*	Generate OH	Broad-spectrum antimicrobial agents for non-antibiotic disinfection are used in biomedicine	Yang et al. (2021)
AuNCs@PCN	POD	*E. coli*, *S. aureus*, MRSA, Amp^r^ *E. coli*	Generate ROS	Promote the wound healing of diabetic infection	Zhao et al. (2022)
ZIF8/Au-GOx NPs	POD	*E. coli*, *S. aureus*	Generate ROS, Release of Zn^+^	High-efficient sterilization and fast promote wound healing	Wang et al. (2022)
Bi-PCN222	OXD, POD	*S. aureus*, MRSA	Generate ROS, electron transport chain	Effective disinfection and tissue reconstruction	Wu et al. (2023)
ZFMs	POD	ESBL-producing *E. coli*	Generate ·OH	Biomedicine	Zhong et al. (2023)
PM@MIL-88B-Fe/Zn	POD	*E. coli*, *S. aureus*	Generate ROS	Accelerate healing of infected wounds	Shi et al. (2023)
UsAuNPs/MOF	POD	*E. coli*, *S. aureus*	Generate ROS	Accelerate the clinical application of nanocatalytic antibacterial therapy	Hu et al. (2020)
MOF@COF	POD	*E. coli*, *S. aureus*	Generate OH	Disease treatment	Zhang R. et al. (2021)
DSAM	SOD, POD, GPx	MRSA, *P. aeruginosa*	Generate OH	Eliminates bacteria and relieves persistent inflammation	Mo et al. (2023)

**FIGURE 6:**
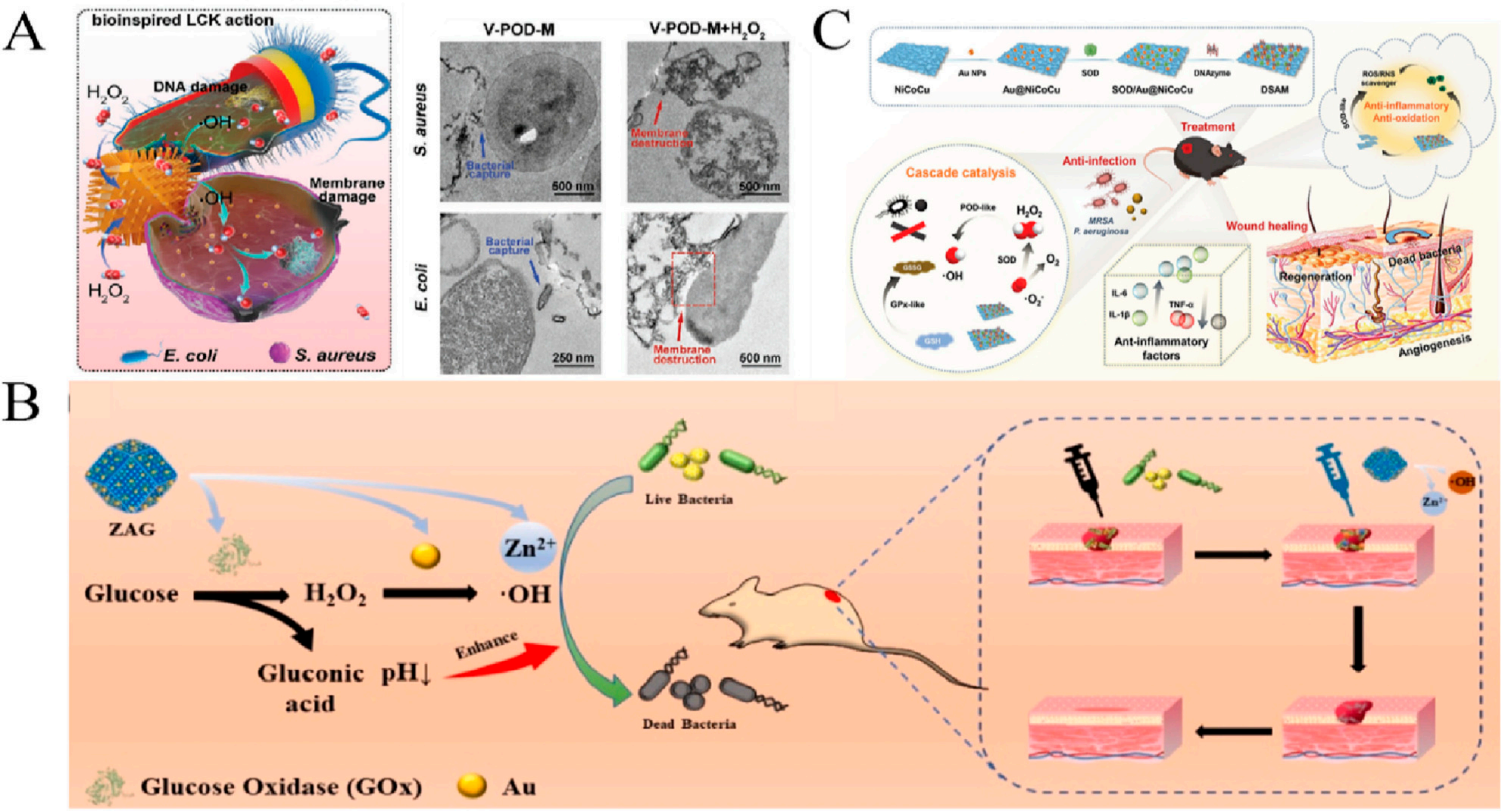
**(A)** Antibacterial properties of V-POD-M (Yang et al., 2021). **(B)** Antibacterial application of DSAM (Wang et al., 2022). **(C)** Antibacterial mechanism and application of ZAG (Mo et al., 2023).

Yang et al. (2021) first reported the synthesis of a virus-like peroxidase mimic (V-POD-M) for efficient bacterial capture and synergistic catalytic sterilization (Figure 6A). Cu (II) MOFs was used as POD to simulate the generation of ·OH radicals, while MoO_3_ was used as auxiliary catalyst to modify MOFs to reduce the energy barrier required for ROS generation and achieve low dose administration. The silicon dioxide was then coated in MOFs to enable the nanozyme to capture bacteria quickly and to sterilise them efficiently at low doses. This provides a promising broad-spectrum therapy for non-antibiotic disinfection. Zhao et al. (2022) introduced Au NCs into PCN-224 by *in situ* growth method and constructed a MOF nanozyme AuNCs@PCN with photocatalytic and antibacterial activity. AuNCs@PCN has excellent POD-like activity and can catalyze the production of ·OH and O_2_ at the wound site of high concentrations of endogenous H_2_O_2_ for chemical kinetics therapy (CDT) and enhanced photodynamic therapy (PDT), it performed well in photothermal therapy (PTT). Under the irradiation of NIR laser, AuNCs@PCN can be heated to 56.2°C and produce ROS, which has obvious bactericidal effect on bacteria and can eradicate bacterial infection and promote wound healing in diabetes mellitus. As shown in Figure 6B, Wang et al. (2022) integrated GOx and Au nanozyme into ZIF8 to prepare ZIF8/Au-GOx NPs (ZAG NPs), developing an acid-enhanced bimodal antimicrobial therapy strategy. ZAG NPs reduced the acidic environment of the wound infection zone through a cascade of catalytic reactions, and also produced ROS, which cooperated with the release of Zn^2+^ for highly effective antibacterial activity. Wu et al. (2023) formed Bi-PCN222, a bionic enzyme catalyst with Schottky heterojunction, by *in situ* reduction of Bi-doped Bi nanoparticles in the metal-organic framework (MOF) of PCN-222. The enzyme not only has oxidase-like and Peroxidase activity, but also has rapid and efficient suicide and wound healing properties.On the one hand, after the bacteria attached to Bi-PCN-222, the bacteria obtained electrons on Bi-PCN-222, which interfered with the respiratory and metabolic pathways of bacteria and enhanced the oxidative stress in bacteria. On the other hand, the electrons on Bi-PCN-222 flow spontaneously to PCN-222, making Bi-PCN-222 have bionic enzyme activity, which effectively catalyzes the production of ROS (^.^OH, O_2_^.-^) by O_2_ and H_2_O_2_. It can be widely sterilized so as to achieve high-efficient and rapid treatment of infected wounds. Zhong et al. (2023) prepared a zinc-regulated Fe-MOF (ZFMs) nanozyme by solvothermal method to promote wound healing of bacterial infection. ZFMs has excellent POD-like activity, and trace amounts of ZFMs can cause 98% lethality to β-lactam producing *Escherichia coli* (ESBL-producing *E. coli*). In addition, ZFMs is a promising wound dressing with long-term stability under physiological conditions (pH 7.4) and good biocompatibility. It is worth noting that the targeting and toxicity of nanozymes are not to be ignored. Taking advantage of the high biocompatibility and targeting properties of the platelet membrane, we have developed a bio-organic nanozyme, PM@MIL-88B-Fe/Zn, which encapsulates a bimetallic organic framework (MIL-88B-Fe/Zn) in the platelet membrane for efficient antimicrobial therapy (Shi et al., 2023). The introduction of Zn accelerated the electron transfer and enzyme kinetics of Fe(III)/Fe(II), resulting in enhanced POD-like activity of MIL-88B-Fe/Zn. Platelet membrane encapsulation innovatively enhances the targeting of nanozyme to bacteria. PM@MIL-88B-Fe/Zn uses its POD activity to catalyze the production of ROS, which effectively inhibits bacterial growth and leads to cell death. In addition, *in vivo* experiments showed that the nanozyme could accelerate the healing of infected wounds. Importantly, PM@MIL-88B-Fe/Zn exhibited superior biodegradability, good metabolism and non-toxic accumulation.

As a kind of ultra-thin two-dimensional nanomaterials, two-dimensional MOFs nanomaterials have better catalytic activity, and are also widely used in antibacterial fields. Using ultrathin 2D MOFs as template, Hu et al. (2020) grew UsAuNPs *in situ* to obtain UsAuNPs/MOFs nanozyme, which showed excellent POD-like activity. The enzyme also showed significant antibacterial activity against both Gram-negative (*E. coli*) and Gram-positive bacteria (*S. aureus*) with the assistance of low-dose H_2_O_2_. Besides, it has good biocompatibility and can promote wound healing effectively. Yang et al. (2024) reported a novel strategy for driving a cascade of enhanced peroxidase (POD) reactions by constructing a 2D conjugated metal-organic framework (MOF) with noncoordination saturation as a nanoenzyme.It is demonstrated that the non coordination saturation Cu atom promotes the adsorption of H_2_O_2_, followed by the Cu-HHTP MOFs with high conjugated structure can enhance the photogenerated electron transfer for ^.^OH generation in POD reaction.The sequential enhancement of substrate adsorption and decomposition endows the as-made Cu-HHTP MOFs with outstanding antibacterial property. Zhang R. et al. (2021) designed and constructed a naturally excited MOF@COF nanozyme with high Peroxidase activity. In addition, it is a nanozyme with an active site in a specific environment and a pseudopodal structure. Not only can effectively capture bacteria, but also *in situ* production of ROS to kill bacteria, with excellent catalytic bactericidal performance, can promote wound healing. Interestingly, Sun’s team reported that an ultra-thin three-metal 2D MOFs sheet nanozyme (Ni_2_Co_1_)_1-x_Cu_x_ (Sun et al., 2020; Lin et al., 2022), which exhibits excellent POD performance. It can catalyze H_2_O_2_ to produce toxic ^.^OH, which can destroy bacteria and effectively promote the healing of infected wounds. However, a higher dose of (Ni_2_Co_1_)_1-x_Cu_x_ is required for antimicrobial action and has no antioxidant activity. Subsequently,2D NiCoCuMOFs(DNAzyme/SOD/Au@NiCoCu MOFs, called DSAM) were constructed by integrating SOD, DNAzyme and Au NPs into 2D nanozymes (Mo et al., 2023). As shown in Figure 6C, DSAM has a variety of enzyme-like catalytic activities, can effectively eliminate bacteria and reduce inflammatory reaction. The nanozyme can utilize the antioxidant property of SOD to supplement O_2_ and H_2_O_2_, and alleviate anoxic micro-environment. In addition, the H_2_O_2_ produced during the reaction kills the bacteria by breaking down the highly toxic ^.^OH through POD-like activity. At the same time, GPx-like activity can deplete GSH and prevent ^.^OH loss. Importantly, the results of antibacterial experiments *in vitro* showed that only 40 μg mL^−1^ of DSAM was needed to achieve the high efficacy of killing MRSA. The bactericidal concentration of (Ni_2_Co_1_)_1-x_Cu_x_ was three times that of DSAM. In addition, the results of wound healing experiments also showed that DSAM has excellent antibacterial, anti-inflammatory, antioxidant and biosafety properties. It can be effective treatment of infected wounds.Wang et al. (2024) prepared a biomedical material (Ce-BDC@Au MOFs) for *in situ* deposition of spherical gold nanoparticles (Au NPs) on a rough surface of Ce (III) and terephthalate acid, with both ROS elimination capability and ROS independent antibacterial capability.Ce-BDC@Au MOFs show good photothermal conversion efficiency under NIR laser (808 nm) irradiation. Benefitting from rough surface and photothermal conversion ability, Ce-BDC@Au MOFs have high antibacterial efficiency against *staphylococcus aureus* through both mechanically damaging and photothermal destruction. This strategy is biosafety and effectiveness for treating diseases related to both ROS accumulation and bacterial infections.

### 3.6 Single-atom nanozymes

Single-atom nanozyme is also a kind of nanomaterials with enzyme-like activity, which has developed rapidly in recent years. In addition to the intrinsic enzyme-like activity of the general nanozymes, the single-atom nanozyme activity center has a high atomic utilization rate and a clear active center, which can be targeted to regulate its activity and selectivity, to ensure that it has better catalytic activity, selectivity and stability. Table 6 lists some of the applications of single-atom nanozymes with enzyme-like activity in antibacterial field.

**TABLE 6:** Application of Single-atom nanozymes in antibacterial.

Nanozymes	Enzymatic activity	Targets	Antibacterial mechanisms	Applications	References
Cu-N-C	OXD, POD	*E. coli*, *S. aureus*, *P. aeruginosa*, *B. subtilis*, MRSA	Generate ROS	It accelerates the death of bacteria, promotes wound healing and can be used clinically	Zhu et al. (2022)
Au@CuBCats	POD	ESLP *E. coli*, MRSA	Generate OH	Treatment of multi-drug resistant bacterial diabetic ulcer	Fan et al. (2023)
Cu_L_/PHI	POD	*E. coli*, MRSA	Generate ROS	Photocatalytic sterilization	Ou et al. (2023)
PMCS	POD	*P. aeruginosa*	Generate OH	Biocatalysis	Xu et al. (2024)
FeSAs@Sa.M	OXD	MRSA	Generate ROS	Treat intracellular infections	Liu et al. (2023)
Fe-N-C	POD	*E. coli*, *S. aureus*	Generate ROS, PTT	Combined antibacterial activity was carried out by photothermal treatment-assisted catalysis	Feng et al. (2022)

**FIGURE 7:**
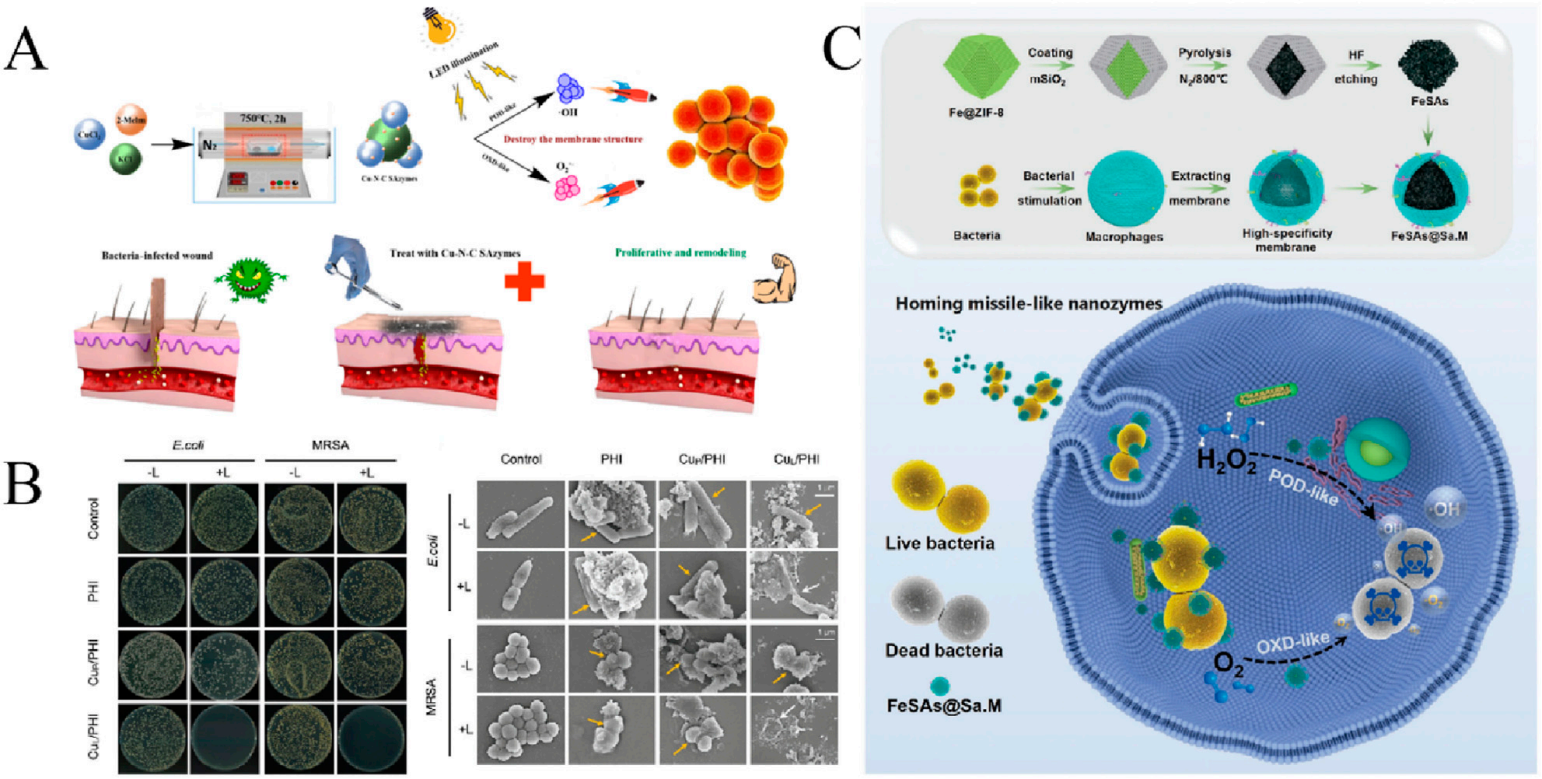
**(A)** Antibacterial effect and application of Cu-N-C (Zhu et al., 2022). **(B)** Antibacterial effect of Cu_L_/PHI (Ou et al., 2023). **(C)** Preparation of FeSAs@Sa.M and its killing of bacteria in host cells (Liu C. et al., 2023).

Cu-based monatomic nanozymes has been widely studied in the field of antibacterial activity because of its active Cu site as a catalytic center and good biocompatibility. Using a salt-template strategy, Zhu et al. (2022) synthesized Cu-N-C nanozymes (Cu-N-C) with high metal loading, which was rich in active Cu sites. It has good specific OXD and POD activity, and can significantly enhance antibacterial activity by releasing O_2_^·-^ and ·OH. The ROS released can oxidize the lipid membrane and destroy the bacterial membrane, promoting the death of bacteria. At the same time, light-emitting diode photoincubation can further improve the antibacterial activity due to photocatalysis. In addition, it was found that Cu-N-C had excellent inhibitory effect on many kinds of bacteria, and showed amazing performance in slowing down the formation of drug-resistant bacteria. In the wound model, Cu-N-C not only accelerated the death of bacteria but also promoted wound healing (Figure 7A). At the same time, the nanozyme has good biocompatibility and great potential for clinical application. In addition, the design of cascaded catalytic nanozymes to avoid the toxicity of exogenous H_2_O_2_, can play a huge potential in antibacterial applications. Fan et al. (2023) synthesized an AuNP-anchoredcopper monatomic biocatalyst (Au@CuBCats) as a novel GOx-POD dual enzyme mimicking therapeutic platform for bacterial diabetic ulcers. AuNPs can act as GO_X_ mimics and catalyze the oxidation of glucose to form H_2_O_2_; H_2_O_2_ can then be further catalysed to ·OH by mimicking a single copper atom without additional energy input. Notably, the unique copper monoatom coordinated by one oxygen and two nitrogen atoms (CuN_2_O_1_) exhibited better POD catalytic performance than HRP. Au@CuBCats has significant antimicrobial activity against multi-drug resistant strains MRSA and ESLP *E. coli*. In addition, it has excellent therapeutic effect and biosafety for multi-drug resistant bacterial diabetic ulcer. At the same time, the catalytic activity of nanozymes can be adjusted by adjusting the active site of monatomic nanozymes so as to improve the antibacterial activity of nanozymes. Ou et al. (2023) synthesized two kinds of Cu mono-site nanosases in the interlayer (Cu_L_/PHI) and in the plane (Cu_p_/PHI) by adjusting the position of Cu mono-atom on the polyheptadine imine (PHI) nano-platform through different synthetic routes. Compared with Cu_p_/PHI, Cu_L_/PHI nanozyme could promote photoelectron transport and O_2_ Activation, and enhance ROS production. Under visible light irradiation, the photocatalytic activity against MRSA was nearly 100%, and the broad-spectrum antibacterial activity against many bacterial strains was also observed (Figure 7B). This may be of great significance for the further development of monoatomic nanozymes with ideal coordination structure for photocatalytic antibacterial applications.

In addition, it has been found that monatomic nanozymes containing other metal active sites also has excellent catalytic activity and broad-spectrum antibacterial properties. Xu et al. (2024) first used zinc-based zeolite-imidazole framework (ZIF8) as a precursor to prepare a highly efficient monatomic nanozyme with excellent Peroxidase activity through a mesoporous silicon dioxide (mSiO_2_)-protected pyrolysis strategy. The high catalytic activity of PMCS nanozymes is attributed to the coordination-unsaturated zinc monatomic active center, which leads to H_2_O_2_ decomposition and ·OH formation. Therefore, it has excellent antibacterial activity against *P. aeruginosa*. At the same time, the enzyme has excellent biological safety, has good therapeutic effect on infected wounds *in vivo*, and can significantly promote wound healing. Liu et al. (2023) developed a homing missile-like nanotherapy (FeSAs@Sa.M), FeSAs@Sa.M is made from a single-atom Fe nanozyme (FeSA) core, which is surrounded by the membrane of a macrophage (Sa.M) infected with MRSA. Mechanically, FeSAs@Sa.M was originally through SA. The bacterial recognition ability of M component binds to extracellular MRSA. Subsequently, the FeSA core, with its high POD and OXD-like activity, can catalyze the production of highly toxic ROS, effectively killing intracellular bacteria (Figure 7C). This work provides new insights into targeted and non-antibiotic antimicrobial therapy. Gao et al. (2024) reported in the rational integration of Mn nanocinases into 3D printed bioceramic scaffold (Mn/HSAE @ BCP scaffold), the integrated Mn/HSAE@BCP scaffold can catalyze the conversion of H_2_O_2_, to generate hydroxyl radical (OH) and superoxide anion (O_2_^.−^).The synergistic strategy of chemical kinetic therapy (CDT)/acoustic dynamics (SDT) produces enough reactive oxygen species (ROS) to kill *S. aureus* (*S. aureus*) or *E. coli* (*E. coli*). At the same time, this study also provides an effective method for the treatment of clinical infectious bone defects. Tao et al. (2024) built a pH-responsive bifunctional platform with multiple enzyme-like catalytic activities for synergistic tumor therapy by integrating Rh single atoms nanozymes (SA-Rh nanozymes) with photothermal therapy (PTT).SA-Rh nanozymes exhibit peroxidase-mimicking activity in tumor cells, inducing a synergistic effect of enhanced reactive oxygen species generation, thereby enabling synergistic cancer therapy involving chemokinetic therapy and PTT.

### 3.7 MXene-based nanozymes

MXenes is a kind of two-dimensional transition metal carbide, nitride or carbonitride nanomaterials with two-dimensional layered structure. Most MXene compositions are composed of C, N and transition metals (Ti, Nb, Ta) that are non-toxic to biological tissues and therefore have good biocompatibility. It was found that MXenes had large surface area, chemical activity and functionalization feasibility, and could load different antibacterial functional groups. Table 7 lists some of the applications of MXene-based nanozymes with enzyme-like activity in antibacterial field.

**TABLE 7:** Application of MXene-based nanozymes in antibacterial.

Nanozymes	Enzymatic activity	Targets	Antibacterial mechanisms	Applications	References
M@P@Lyso	—	MRSA	PTT, Electrostatic interaction, Lysozyme catalysis	Stimulate the enzyme nano-platform to address bacterial resistance	Zhang D. et al. (2023)
Nb_2_C@Gel	—	*E. coli*, *S. aureus*	PTT	Treatment of diabetic wounds	Chen J. et al. (2022)
V_2_C NSs	—	*E. coli*, *S. aureus*	PTT, Physical damage	Rapid sterilization for biomedical and industrial applications	Geng et al. (2023) Zada et al. (2021)
V_2_C MXene nanosheets	OXD	*E. coli*, *S. aureus*	Generate ROS	It opens up a new way for the further development and design of bacterial environmental colorimetry	Lian et al. (2023)
Ag-MXene	POD	*E. coli*, *S. aureus*	Generate OH	Rapid detection and sterilization in the field of biomedicine	Chen et al. (2024)

**FIGURE 8:**
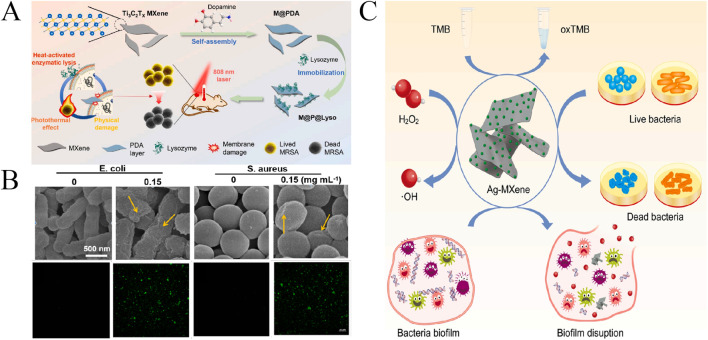
**(A)** Preparation of the M@P@Lyso and their photothermal-enhanced antibacterial activity (Zhang D. et al., 2023). **(B)** Antibacterial effect of V_2_C MXene nanosheets (Lian et al., 2023). **(C)** Antibacterial perfoemence of Ag-MXene (Chen et al., 2024).

The antibacterial behavior of Ti_3_C_2_T_x_ MXene was first reported by Rasool et al. (2016), who experimentally found that the sharp edges of two-dimensional Ti_3_C_2_T_x_ nanosheets damaged the bacterial cell wall, both Gram-negative *E. coli* and Gram-positive *B. subtilis* showed excellent antimicrobial activity in a dose-dependent manner. Since then, it has also focused on Ti_3_C_2_T_x_ and its composites, for example, Zhang D. et al. (2023) have chemically modified Ti_3_C_2_T_x_ MXene nanosheets with a polydopamine (PDA) surface to enhance photothermal effects and performance durability, M@P@Lyso was synthesized by immobilization of lysozyme biomacromolecules on the two-dimensional hybrid interface via intermolecular electrostatic affinity. As shown in Figure 8A, M@P@Lyso effectively inhibited the proliferation of MRSA with negligible cytotoxicity and excellent biocompatibility. It should be noted that the outstanding antibacterial activity of M@P@Lyso is due to the synergy of multiple bactericidal mechanisms, that is, the photothermal enhancement of lysozyme biocatalytic activity, the local overheating effect produced by M@PDA, and the physical cleavage effect of the nanoplates.There are also a number of other phases with excellent antibacterial properties of MXenes have been reported in the antibacterial field. Shuai et al. (2024) designed the Ti_2_C_3_ nanosheet/tin disulfide (MXene/SnS_2_) heterojunction, which integrates photothermal and photodynamic properties.Then, MXene/SnS_2_ was incorporated into a poly-L-lactic acid powder (PLLA) matrix to fabricate an artificial bone scaffold with selective laser sintering (SLS) technology. Under near-infrared laser irradiation, SnS_2_ can strengthen the near-infrared light absorption of MXene to generate local hyperthermia, thus enhancing bacterial membrane permeability. Meanwhile, MXene/SnS_2_ enhances charge transfer and inhibits electron-hole pair separation, thereby generating more ROS that can penetrate the bacterial interior. The results indicated that this antibacterial strategy has effective antibacterial activity. (Chen P. et al., 2022). developed an injectable heat-sensitive Nb_2_C-based hydrogel (Nb_2_C@Gel) with antioxidant and antibacterial activities. *In vitro* experiments have shown that Nb_2_C NSs has good photothermal conversion capacity and can achieve efficient bactericidal efficacy through synergy of intrinsic antibacterial activity and photothermal bacterial ablation. Nir-mediated PTT combined with Nb_2_C@Gel could eliminate bacterial infection and reduce inflammatory reaction and oxidative stress induced by infection at low temperature. Zada et al. (2021) prepared 2D V_2_C nanosheets (V_2_C NSs) with high yield by an environmentally friendly and low-cost delamination method. The resulting V_2_C NSs had good structural reliability and inherent antibacterial ability, and at a concentration of 80 μg mL^−1^, the V_2_C NSs was able to efficiently disrupt the bacterial cell membrane by disrupting the cell membrane, it kills Gram-positive bacteria *S. aureus* and Gram-negative *E. coli*. Furthermore, upon near-infrared laser irradiation, the photothermal effect combined with the intrinsic antibacterial ability of V_2_C NSs exhibited excellent antibacterial ability at low concentrations (40 μg mL^−1^) of V_2_C NSs and near-infrared irradiation, the killing rate of the two bacteria in 4 h was over 99.5%. In addition, we synthesized V_2_C MXene by simple intercalation and exfoliation method (Lian et al., 2023). The results showed that the prepared V_2_C MXene had excellent OXD catalytic activity, it can promote the catalytic oxidation of O_2_ to produce ROS without H_2_O_2_. Due to its special reactive oxygen species production capacity, V_2_C MXene nanosheets have significant broad-spectrum antibacterial activity against Gram-positive and Gram-negative bacteria (Figure 8B). Compared with the effect of pure MXene in biomedicine application, the combination of MXene with other nanomaterials can improve the catalytic and antibacterial properties of MXene. As shown in Figure 8C, Chen et al. (2024) used a simple *in situ* reduction strategy to prepare Ag-MXene nanozyme with excellent POD activity. In the presence of H_2_O_2_, it can produce ^.^OH, which has good antibacterial activity against both *S. aureus* and *E. coli*. In addition, the binding of Ag-MXene to H_2_O_2_ also disrupts the formation of bacterial biofilms. Jin J. et al. (2024) prepared a magnetic MXene @Fe_3_O_4_/PDA nanosheet with photothermal and magnetic coupled antibacterial properties. Fe_3_O_4_ was grown *in situ* on MXene nanosheets and then coated with a layer of polydopamine (PDA). MXene@Fe_3_O_4_/PDA nanosheet (120 μg mL^−1^) showed an over 95% inhibition rate against *E. coli* and *S. aureus* under 808 nm laser irradiation.

### 3.8 Other nanozymes

In addition to the above nanozymes, there are some other nanozymes (Table 8) also have high catalytic activity and antibacterial properties, widely used in many fields.

**TABLE 8:** Application of Multifunctional nanozymes in antibacterial.

Nanozymes	Enzymatic activity	Targets	Antibacterial mechanisms	Applications	References
Fe_3_O_4_@MOF@Au NPs (FMA NPs)	POD	*E. coli*, *S. aureus*	Generate OH	Healing of bacterial wound infection	Liu et al. (2023)
CuS@Pt-Au/Apt NPs	POD	*E. coli*, *S. aureus*	Generate OH, PTT, CDT	Multimodal antimicrobial therapy for chronic wound infection	Zhang D. et al. (2023)
Ti_3_C_2_ MXene/Fe-MOFs composite (MXM)	POD	*E. coli*, C. Albicans, MRSA	Generate ROS, PTT, CDT	To treat MDR bacterial infection and promote wound healing	Zhao et al. (2024)
BC-fibers	POD, CAT	*E. coli*, *S. aureus*	Generate OH	Provide a way for the integration of incompatible nano-enzymes and broadens the application potential of multi nanozymes	Li G. et al. (2022)
MoS_2_-hydrogel	POD	*E. coli*, *S. aureus*	Generate OH, PTT	Reduces inflammation and promotes wound healing	Sang et al. (2019)
Gel	POD	*E. coli*, *S. aureus*	Generate ROS	Treat bacterial infections and promote wound healing	Qiu et al. (2020)
MOF(Fe-Cu)/GOx-PAM	POD	*E. coli*, *S. aureus*	Generate ROS	Wound healing and clinical application	Tian et al. (2023)

**FIGURE 9:**
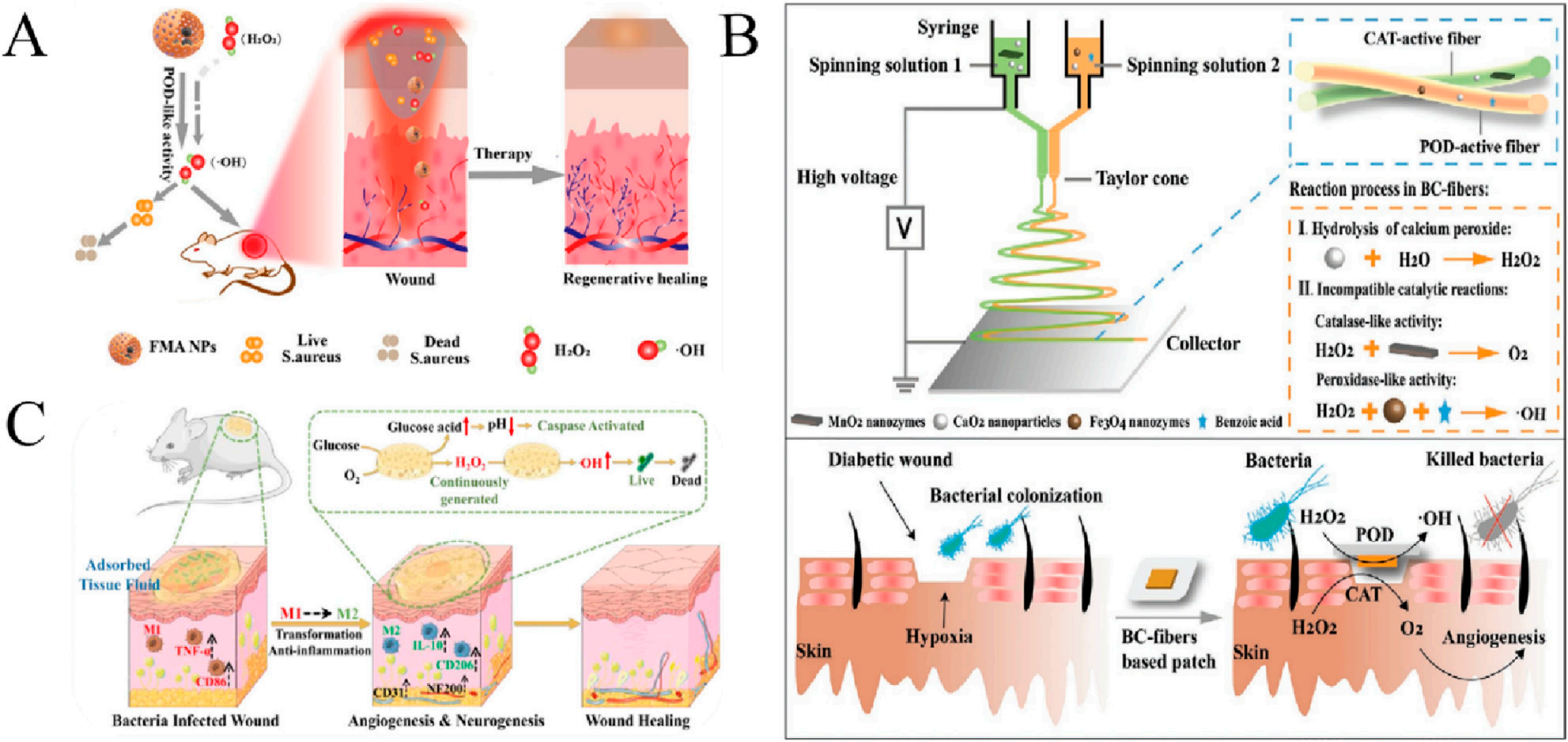
**(A)** Application of FMA nanozyme with POD-like property in antibacterial *in vivo* (Liu et al., 2023). **(B)** The design principle of BC-fibers and the mechanism of accelerating wound healing by synergistic catalytic therapy (Li A. et al., 2022). **(C)** Mechanism of MOF(Fe-Cu)/GO_X_ hydrogel dressings in promoting wound healing (Tian et al., 2023).

In order to better and more effective sterilization, there are many reports of complex nanozyme with more efficient catalytic activity. It can remove bacteria quickly and efficiently, and effectively treat bacterial infection. At the same time, it can also improve the stability and biological safety of nanozymes. For example, Liu et al. (2023) designed a novel hybrid nanozyme with high enzyme catalytic activity and antibacterial activity for wound healing (Figure 9A). Fe_3_O_4_@MOF@Au NPs (FMA NPs) were successfully prepared by functionalizing Fe_3_O_4_ with porous MOF (IRMOF-3) and integrating Au NPs into IRMOF-3 by *in situ* growth. The cascaded catalytic reaction of Au and Fe_3_O_4_ NPs resulted in a synergistic enhancement of POD activity, which further improved the antibacterial efficiency of FMA NPs and could achieve a high antibacterial therapeutic effect in the presence of low concentration of H_2_O_2_. In addition, the dispersion stability and biosafety of Fe_3_O_4_ nanoparticles were improved by using IRMOF-3 nano-shell to coat the surface of Fe_3_O_4_ nanoparticles. Therefore, the nanozyme has great potential for healing bacterial wound infection. In order to achieve both accurate targeting and efficient sterilization, Zhang Y. et al. (2023) successfully constructed a novel aptamer-functionalized near-infrared light-responsive antibacterial nanomaterials (CuS@Pt-Au/Apt NPs) by a simple nano-precipitation method, for the treatment of wound infections. CuS@Pt-Au/Apt NPs can specifically target bacterial surfaces, combined with the hyperthermia induced by near infrared radiation, the sustained release of hydroxyl by high concentration of glucose cascade nanozyme in diabetic wound and the destruction of antioxidant defense system by Cu^2+^ produced by CuS, this causes irreversible damage to the bacteria. This strategy solves the limitation of nanozymes application and provides a solid foundation for the treatment of chronic wound infection. Interestingly, Li G. et al. (2022) used electrospinning to integrate two pH-incompatible POD-like and CAT-like nanozymes and developed a fiber-based multipurpose compartmentalization strategy, a multi-nanometer nanozyme system capable of simultaneous incompatible reactions was constructed. As shown in Figure 9B, by controlling the pH of the different fiber compartments, two incompatible nanozymes can act simultaneously in their respective preferred microenvironments and each exhibits optimal catalytic activity. The system activates rapidly in the presence of water, releasing both O_2_ and ^.^OH, showing a synergistic ability to reduce hypoxia and kill bacteria. Further *in vivo* experiments showed that this system could accelerate the synergistic healing of diabetic wounds.In addition, hydrogel nanozymes with antibacterial function is one of the research hotspots in the field of biomedicine in recent years. For example, Sang et al. (2019) constructed a positively charged porous MoS_2_-hydrogel for effective antibacterial activity. The antibacterial effect of ^.^OH was effectively enhanced by trapping and limiting the bacteria to ROS damage by electrostatic interaction. In addition, 808 nm laser irradiation can produce synergistic antibacterial effect. More importantly, nanozyme-hydrogels not only reduce the incidence of inflammation but also promote wound healing by removing dead bacteria from the wound site. Qiu et al. (2020) prepared supramolecular hydrogels by self-assembly of copper and L-Aspartic Acid (L-Asp). The hydrogel has an intrinsic POD-like catalytic activity that converts H_2_O_2_ to highly toxic ROS and has broad-spectrum antibacterial activity against drug-resistant Gram-positive and Gram-negative bacteria at low H_2_O_2_ levels. At the same time, copper ions can be released slowly from the hydrogel, accelerate the wound site cell proliferation, promote the healing rate, effectively promote the treatment of bacterial infection wound. Tian et al. (2023) constructed a hydrogel wound dressing consisting of a bimetallic MOF loaded with glucose oxidase (GOx). Firstly, MOF(Fe-Cu) with POD property was obtained by classical hydrothermal method, and then GOx was loaded onto MOF(Fe-Cu) by physical adsorption to construct a self-activation cascade reaction system MOF(Fe-Cu)/GOx-PAM gel dressings were prepared by polymerization of MOF(Fe-Cu)/GOx with acrylamide (AM) and Bis-AM. This cascaded catalytic system, through GOx decomposition of glucose, can be continuously generated rich gluconic acid and H_2_O_2_. The gluconic acid can significantly improve the peroxidase properties of MOF(Fe-Cu), further efficiently decompose H_2_O_2_, achieve the antibacterial effect, and promote the regulation of inflammation, thus accelerating the healing of infected wounds (Figure 9C).

## 4 Nanozymes combined with external stimulation antibacterial

To continuously improve the catalytic efficiency, stability and antimicrobial potency of nanoenzymes, researchers are working to innovate and fabricate nanoenzymes with a range of enzyme functions. They are also exploring synergistic antibacterial strategies to create multifunctional nanoenzymes. This work aims to endow these nanoenzymes with enhanced antimicrobial capabilities for the rapid and effective management of bacterial infections. Based on the above detailed description of the progress of different types of nanoenzymes in antimicrobial research, we will focus on outlining the applications of selected nanoenzymes and their integration with light and sound technologies.

### 4.1 Antibacterial activity of nanozymes combined with photothermal therapy

Photothermal therapy (PTT) is a minimally invasive technique based on photochemical reactions that convert light energy, usually near-infrared light, into heat energy. The combination of PTT and nanozymes can improve the efficiency of photothermal transformation and the catalytic activity of nanozymes, which is effective in the treatment of bacterial infection and the avoidance of drug resistance. In recent years, the combination of PTT and nanozymes to play a synergistic antibacterial effect has also attracted extensive attention of researchers. For example, Guo et al. (2022) successfully constructed a photothermal-enzymes catalytic synergistic antibacterial platform. The synthesized hollow mesoporous IONPs possess excellent biosafety, photothermal conversion and peroxidase catalytic activity. It can induce H_2_O_2_ to catalyze the production of ·OH, and have obvious killing effects on *E. coli* and *S. aureus*. At the same time, it can effectively promote the healing of *S. aureus* infected skin wounds and has great potential in clinical anti-infective treatment. Liu et al. (2021) designed a carbon-iron oxide nanocomposite with a rough surface (RCF) for NIR-II photoresponsive synergistic antibacterial therapy. RCF exhibits excellent photo-thermal properties and peroxidase catalytic activity under NIR-II irradiation, and has enhanced antibacterial efficiency to achieve the elimination of *E. coli*, *S. aureus* and MRSA. In addition, satisfactory biocompatibility can be achieved *in vivo* synergistic antibacterial treatment of drug-resistant bacterial infections. Zhang L. et al. (2021) prepared Au-Pt nanodots (AuPtNDs) by one-step synthesis. Under the irradiation of 808 nm Laser, AuPtNDs has good photo-thermal conversion and strong photo-thermal stability. It not only exhibits strong peroxidase activity, but also has a higher affinity for H_2_O_2_. It can also be used in combination with chemical kinetics to fight bacterial infections and has a broad spectrum of antibacterial properties. Li J. et al. (2021) synthesized a dopamine-coated Iron(II) sulfide therapeutic platform (FeS@PDA). PTT not only inactivates bacteria at high temperatures, but also accelerates the Fenton reaction to produce more hydroxyl radicals. It has excellent broad-spectrum antibacterial activity. It can also effectively treat full-thickness skin defect caused by *S. aureus* infection and accelerate wound healing. Feng et al. (2022) designed a spherical mesoporous Fe-N-C single-atom nanozyme to enhance the Fenton-like catalytic process by photo-thermal treatment. In the light, showed excellent near-infrared absorption and bloom heat conversion efficiency, enhanced catalytic activity. Due to the synergistic effect of nanozyme catalysis and photothermal treatment, the antibacterial activity is significantly enhanced, which can effectively kill bacteria on infected wounds and accelerate wound healing.

To summarise, the integration of nano-enzymes with photothermal therapy is a cutting-edge strategy for antimicrobial chemotherapy. The combination of the two provides a targeted, minimally invasive, and highly effective means of combating microbial infections. At the same time this approach offers a promising and sustainable programme for significantly advancing our fight against drug-resistant bacteria.

### 4.2 Antibacterial activity of nanozymes combined with photodynamic therapy

Photodynamic therapy (PDT) is a new technique for diagnosis and treatment of diseases using photodynamic effects. Photosensitizers are irradiated by a specific wavelength of laser, and the photosensitizers are stimulated to produce toxic ROS, to achieve the elimination of bacteria. PDT is fast, efficient and easy to operate. When nanozymes are incorporated into this therapeutic modality, they augment the ROS production, leading to a more pronounced oxidative stress on the bacteria. This synergistic effect not only intensifies the bactericidal action but also broadens the spectrum of microbial targets, including antibiotic-resistant strains.The precision of nanozymes, with their ability to be engineered for specific catalytic activities, combined with the spatial and temporal control of light exposure in PDT, allows for a highly targeted and efficient antimicrobial intervention. It has been reported that the size of nanomaterials will affect their catalytic and antibacterial properties and may also have an impact on PDT capabilities. Xue et al. (2023) first reported the effect of nanomaterials size on PDT properties. Two-dimensional porphyrin-based PCN-134 MOF nanoparticles were synthesized by a two-step solvothermal method for enhanced photodynamic antibacterial therapy. Two-dimensional PCN-134 nanosheets with different transverse sizes and thicknesses were successfully prepared by controlling the reaction temperature. It was found that the photodynamic activity of PCN-134 nanoplates increased with the decrease of the size of PCN-134 nanoplates irradiated by 660 nm Laser. The 2D small PCN-134 nanosheets (S-PCN-134) have higher catalytic activity for ROS generation under 660 nm laser irradiation. Therefore, PVP@S-PCN-134 nanosheets can be used as photodynamic antibacterial agents after PVP modification. The results of *in vitro* and *in vivo* experiments showed that PVP@S-PCN-134 nanoplates can effectively destroy bacteria and heal wounds under 660 nm laser irradiation. At present, many studies have been carried out to prepare new nanomaterials for antibacterial application by combining catalytic activity with PDT. Li W. et al. (2023) designed a kind of Polyethylene glycol ointment (CuTCPP-Fe_2_O_3_) with Fe_2_O_3_-modified by atomic layer in a two-dimensional porphyrin-based MOF (CuTCPP). CuTCPP-Fe_2_O_3_ heterojunction has good biocompatibility and biodegradability. Under the synergistic action of reactive oxygen species and released ions, the bacteria exhibited broad-spectrum antibacterial activity (>99%) against a variety of oral pathogens, including P. gingivalis, F. nucleatum and *S. aureus*. This kind of photodynamic ion therapy has shown excellent therapeutic effect in the treatment of periodontitis reported in the clinic. The Ag/Bi_2_MoO_6_ (Ag/BMO) nanozyme with photoactive and sustained Peroxidase activity and NIR-II photodynamic properties was prepared by charge separation engineering (Jiang et al., 2018). Ag/BMO nanozyme had excellent photo-enhanced enzyme-like activity and the ability of producing ^1^O_2_ and ·OH continuously. Ag/BMO nanozyme had good bactericidal activity (∼99.9%) against MRSA. Theoretical calculations show that the introduction of Ag into BMO makes it easier for phototriggered electron-hole pairs to separate and produce ROS. Under the irradiation of 1064 nm Laser, the electron transfer to BMO is beneficial to the reversible change of Mo^5+^ Mo^6+^, which improves the catalytic activity of peroxidase and the photodynamic properties of NIR-II. In addition, the excellent antibacterial activity of Ag/BMO NPS was related to the peroxidase activity, the photodynamic behavior of NIR-II and the acidic enhancement of Ag^+^ release. Based on this excellent performance, the nanozyme can be used as an alternative antibiotic to treat infectious diseases. In addition, in order to target bacteria, effective and accurate elimination of bacterial infection. Li Z. et al. (2023) constructed a novel nanozyme, S-MM@CeO_2_-TCPP, which was composed of mesoporous CeO_2_ nanospheres and meso-tetra(4-carboxyphenyl)porphine (TCPP) encapsulated in pathogen-activated macrophage membrane. Mesoporous CeO_2_ hollow nanospheres were used as carriers. After TCPP was introduced, the nanozyme could not only play the role of PDT, but also enhance the POD-like enzyme activity of CeO_2_ by photocatalytic mechanism. The encapsulation of pathogen-activated macrophage membranes endows the nanozyme with high expression of Toll-like receptors, enabling it to target bacteria. PDT combined with nanozyme synergizes with light-enhanced CDT, and nanozyme-targeted aggregation at the site of infection produces large amounts of ROS, which effectively kills bacteria and reduces inflammation, promoting wound healing in infection. This strategy also provides a more effective and reliable alternative for the precise elimination of pathogens from microbial-infected wounds and the reduction of inflammation.

In conclusion, the integration of nanozymes with photodynamic therapy represents a paradigm shift in the development of antimicrobial treatments. It offers a non-invasive, highly effective, and adaptable strategy that could significantly reduce the reliance on traditional antibiotics, thereby mitigating the growing threat of antimicrobial resistance. This innovative alliance between nanotechnology and light-based therapies holds the potential to usher in a new era of antimicrobial therapies, providing a robust and sustainable solution to the global challenge of drug-resistant infections.

### 4.3 Antibacterial activity of nanozymes combined with sonodynamic therapy

Sonodynamic therapy (SDT) uses ultrasound (US) to stimulate sonosensitizers to produce reactive oxygen species, which can cause irreversible damage to bacteria and has high broad-spectrum antibacterial activity. At present, SDT is a new technology with high tissue penetration, site-specific ultrasound and good biocompatibility, which is widely used in biological therapy and antibacterial field. In addition, the antimicrobial activity of SDT may be mainly dependent on the mechanical injury effect of ultrasound and the cytotoxic effect of ROS. The production of ROS depends on the nature of the sonosensitizer used. However, most reported sonosensitizers show limited bioavailability and high clearance *in vivo*. Using nanozyme as sonosensitizer combined with SDT can improve the antibacterial activity of bacteria and solve the problem of complex bacterial infection. Sun et al. (2020) designed a US-switchable nanozyme system, Pd@Pt-T790, which was constructed by enzyme-catalyzed bridging of Pd@Pt nanoplates with meso-tetra(4-carboxyphenyl)porphine (T790), an organic sonosensitizer. It can be activated in the process of ultrasound can be controlled catalytic production of oxygen, promote the production of ROS, enhance SDT against deep bacterial infection. Interestingly, the modification of T790 on Pd@Pt significantly blocked its Catalase activity, whereas after US irradiation, the activity of the nanozyme was effectively restored, catalyzing the decomposition of endogenous H_2_O_2_ to O_2_. This reduces the potential toxicity and side effects of the nanozyme on normal tissues. In addition, MRSA-induced myositis can be completely eradicated due to the US-switchable enzyme activity of Pd@Pt-T790, excellent accumulation at the site of infection, and good biocompatibility. In addition, nanozymes can use multi-enzyme activity to set up a cascade catalytic reaction system to meet the needs of bacterial infection treatment. Shang et al. (2023) used hyaluronic acid encapsulated L-arginine, ultrafine gold nanoparticles and Cu_1.6_O to co-load p-doped graphite nitride carbon nanoparticles, a kind of ultrasound-enhanced multi-enzyme-like (SOD-CAT-GOx-POD/NOS) nanozyme hydrogel spray (Au/Cu_1.6_O/P−C_3_N_5_/Arg@HA, denoted as ACPCAH) was developed. Hyaluronic acid entrapment not only improves the biocompatibility and stability of the nanozyme, but also specifically breaks down by hyaluronidase in the biofilm, releasing L-arginine and nanozyme and enhancing the interaction with bacteria. In addition, nanozyme-mediated catalysis and acoustic catalysis can further enhance the antibacterial activity of ACPCAH under ultrasound. *In vitro* and *in vivo* tests showed that ACPCAH has many functions such as anti-inflammation, anti-bacteria, oxygen supply and promoting cell growth. It can eradicate pathogenic bacteria and accelerate wound healing of diabetes mellitus effectively. It is worth noting that nanozyme targeting on bacteria, enhance the specific killing of bacteria, more effective solution to the problem of bacterial infection. Yao et al. (2023) reported a bimetallic BiPt nanozyme for pathogen-targeted nanocatalysis in the treatment of multi-drug resistant pathogen-associated infections *in vivo*. BiPt nanozyme exhibit dual enzyme activities (OXD and POD), which can produce highly toxic ·O_2_^−^, ·O_2_^2−^, and ·OH simultaneously in the inflammatory microenvironment, thanks to the electron coordination effect. In addition, the introduction of ultrasound enhanced the catalytic activity of the two enzymes and the efficiency of ROS production. What’s more, BiPt nanozyme is further covered by a platelet-bacterial hybrid membrane (BiPt@HMVs), which has precise homologous targeting to pathogens. Thus, BiPt@HMVs can eliminate carbapenem-resistant Enterobacterales (CRE) and MRSA by combining precise targeting with efficient catalysis, effectively dealing with infections caused by multi-drug resistant bacteria.

The combination of nanotechnology and Sonodynamic therapy (SDT) represents a pioneering approach in the field of antimicrobial drug therapy. By utilising the catalytic ability of nanoenzymes and the mechanical energy of ultrasound, this synergistic strategy effectively improves the antibacterial effect. The high-frequency vibrations induced by the sound waves disrupt the cell walls of the bacteria, thereby increasing their susceptibility to the enzymatic activity of the nanoenzymes. This dual mechanism of action not only accelerates bacterial inactivation but also minimises the possibility of resistance development. Thus, the combination of nanoenzymes and Sonodynamic therapy (SDT) becomes a powerful and innovative strategy that offers a promising prospect for the development of next-generation antimicrobial therapies that are both effective and environmentally friendly.

## 5 Summary

In conclusion, the different types of nanozymes and their applications in the antibacterial field were introduced. In addition, compared with the single action of nanozymes, the combination of external stimulation (such as light, ultrasound) and the catalytic activity of nanozymes can enhance the broad-spectrum antibacterial activity of nanozymes and have a good therapeutic effect on bacterial infections. Although some achievements and progress have been made in the field of antibacterial activity in recent years, there are still many problems and challenges that need to be solved in the future.

(1) Most nanozymes require H_2_O_2_ to exert antibacterial activity through POD activity, and some nanozymes exhibit antibacterial effects when used in combination with other antibacterial therapies. However, in practical applications, the use of external conditions such as H_2_O, light, and ultrasound is limited. Therefore, it is necessary to develop nanozymes with high catalytic activity in the future, so as to achieve efficient sterilization without resorting to external conditions.
(2) The antibacterial mechanism is unclear. Different nanozymes have different antibacterial mechanisms. Currently, most research focuses on destroying bacteria by generating reactive oxygen species through the activity of nanozymes. In the future, we need to conduct more comprehensive and in-depth research on its mechanism.
(3) The *in vitro* toxicity studies of nanozymes need to be rigorous. Most studies have demonstrated that the cytotoxicity of nanozymes is negligible through *in vitro* model cell toxicity experiments. However, if a large number of nanozymes are needed in practical application, the cumulative toxicity can not be ignored. Therefore, this issue still needs extensive research to ensure that toxicity will not affect the antibacterial effect of nanozymes in practical applications.
(4) The *in vivo* biosafety of nanozymes requires significant attention. Although most reports indicate that nanozymes have high bactericidal efficiency and good biosafety. However, research mainly focuses on evaluating the therapeutic effect of nanozymes on mouse skin infection models, and there are few studies on the treatment of *in vivo* infections. Therefore, more attention should be paid to evaluating the efficacy and biosafety of nanozymes in treating infections *in vivo*.
(5) The targeting of nanozymes to bacteria needs to be improved. Although nanozymes can exert broad-spectrum antibacterial effects, their effects on different bacteria are different. Non-targeted nanozymes may cause side effects on normal tissues and cells and reduce their antimicrobial activity. Therefore, it is necessary to functionally design nanozymes to promote the interaction between nanozymes and bacteria, enhance the specific killing of bacteria by nanozymes, and avoid damage to normal tissues and cells.
(6) Research on the antibacterial properties of nanozymes is mostly concentrated in laboratories. If practical application is to be realized, the large-scale production and cost of nanozymes need to be considered, and the performance of antibacterial nanozymes must be stable, safe and efficient.

In short, nanozymes has been developed rapidly in the field of antibacterial in recent years, but there are still many challenges in practical application. However, we are confident that these issues will be properly addressed in the near future. Designing and developing efficient, safe, drug-resistant, and long-lasting antibacterial nanozymes is the direction that most researchers work together.
